# Wavefront control of subcycle vortex pulses via carrier-envelope-phase tailoring

**DOI:** 10.1038/s41377-023-01328-7

**Published:** 2023-11-24

**Authors:** Yu-Chieh Lin, Katsumi Midorikawa, Yasuo Nabekawa

**Affiliations:** Attosecond Science Research Team, RIKEN Center for Advanced Photonics, 2-1 Hirosawa, Wako, Saitama, 351-0198 Japan

**Keywords:** Ultrafast lasers, Ultrafast photonics

## Abstract

The carrier-envelope phase (CEP) of an ultrashort laser pulse is becoming more crucial to specify the temporal characteristic of the pulse’s electric field when the pulse duration becomes shorter and attains the subcycle regime; here, the pulse duration of the intensity envelope is shorter than one cycle period of the carrier field oscillation. When this subcycle pulse involves a structured wavefront as is contained in an optical vortex (OV) pulse, the CEP has an impact on not only the temporal but also the spatial characteristics owing to the spatiotemporal coupling in the structured optical pulse. However, the direct observation of the spatial effect of the CEP control has not yet been demonstrated. In this study, we report on the measurement and control of the spatial wavefront of a subcycle OV pulse by adjusting the CEP. To generate subcycle OV pulses, an optical parametric amplifier delivering subcycle Gaussian pulses and a Sagnac interferometer as a mode converter were integrated and provided an adequate spectral adaptability. The pulse duration of the generated OV pulse was 4.7 fs at a carrier wavelength of 1.54 µm. To confirm the wavefront control with the alteration of the CEP, we developed a novel $f$-2$f$ interferometer that exhibited spiral fringes originating from the spatial interference between the subcycle OV pulse and the second harmonic of the subcycle Gaussian pulse producing a parabolic wavefront as a reference; this resulted in the successful observation of the rotation of spiral interference fringes during CEP manipulation.

## Abstract (web summary)

We perform wavefront control in a subcycle optical vortex pulse by manipulating its carrier-envelope phase. This adjustment affects both temporal and spatial characteristics through spatiotemporal coupling in the structured pulse.

## Graphical abstract

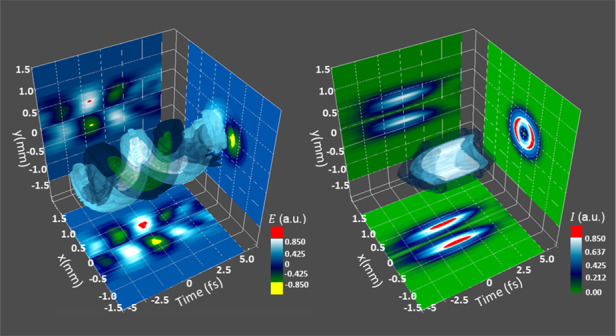

## Introduction

Sculpturing light beams, with full-dimensional controllability of the phase, amplitude, and polarization in both space and time^1,2^, produces pioneering applications in laser manufacturing^3^, long-range quantum communication^4,5^, new forms of multiple moments in matter and free space^6^, and strong-field physics^7–14^. Among the variety of spatiotemporal beams, the “optical vortex” (OV) beam has particularly attracted attention over the past decades because of its interesting feature of carrying the optical orbital angular momentum (OAM)^15^ corresponding to a helical wavefront with a mathematical form of exp (–*iℓϕ*), where $\phi$ is the azimuth angle and *ℓ* is the topological charge or the phase winding number. Owing to this distinct property, the OV beam has been applied in numerous fields and applications, such as laser manipulation/trapping^16^, laser processing^17–20^, quantum information^21,22^, super-resolution microscopy^23,24^, nonlinear spectroscopy^25,26^, and optical communications^27,28^. In current technologies, the generation of a continuous-wave (CW) beam or quasi-CW OV beam has been quite sophisticated; several approaches can be adopted by converting a TEM_00_ Gaussian beam into an OV beam by using astigmatic lenses^29^, spiral phase plates^30^, optical wedges^31^, computer-generated holograms (CGHs)^32,33^, a digital micromirror device (DMD)^34^, and a spatial light modulator (SLM)^35^. However, for a spatiotemporal OV beam, which is accompanied by a broad spectral bandwidth and temporally confined in an ultrashort pulse duration, the generation techniques have challenges associated with chromaticity.

Recently, there has been a significant advancement in the development of these ultrashort pulsed OV beams, which are called the ‘OV pulse’ hereafter. Several achromatic techniques have been reported by utilizing prisms^36,37^, 2f-2f^38^, 4f configurations^39–41^, space-variant waveplates^42,43^, or a Sagnac interferometer (SI)^44,45^. Significantly, the combination of ultrafast amplifier technologies with various types of achromatic methods has shown the possibility of generating OV pulses with ultrashort durations and high output energies^41–43^. This experimental scheme is highly promising for studies on nonlinear phenomena, such as high-order-harmonic generation (HHG)^7–14,46^, filamentation^47,48^, and supercontinuum generation^49^. However, the pulse durations in the reported studies are restricted to multiple optical cycles; the shortest record thus far is 2.3 cycles in the near-infrared (NIR) region^43^. As the pulse duration becomes even shorter than that in the single cycle or equal to that in the subcycle regime, complete control of the spatiotemporal evolution of the electric field in the OV pulses are crucial for studying physical phenomena that directly depend on the electric field rather than the pulse intensity envelope. In this control, the carrier-envelope phase (CEP) is one of the essential parameters that specifies the spatiotemporal characteristic of the subcycle OV pulse. However, there has been no experimental demonstration of the spatiotemporal control of subcycle OV pulses utilizing CEP.

In this study, we demonstrate the generation of subcycle OV pulses and the control of their spatial wavefront by manipulating the CEP. This paper is organized as follows. In Results, we first present the technique and experimental setup used to generate subcycle OV pulses and analyze the spatial wavefront. The subcycle pulse with a Gaussian spatial mode delivered from the optical parametric amplifier (OPA) system, which we developed in our previous study^50^, is converted to the OV pulse by utilizing an SI^44^, which has a large acceptable spectral width. To characterize the rotation of the helical wavefront upon changing the CEP of the subcycle OV pulse, we construct a Mach–Zehnder interferometer (MZI) for measuring the spiral interference fringes between the subcycle OV pulse and the second-harmonic (SH) subcycle pulse in Gaussian mode. This is the extension of the conventional one-dimensional (1D) $f$-2$f$ interferometer for characterizing the CEP of the electric field to the two-dimensional (2D) $f$-2$f$ interferometer for characterizing the CEP-dependent spatial wavefront. We characterize the spatial phase distribution of the OV pulse at five different wavelength components by evaluating its Fermat’s spirals^51^ extracted from the spiral interference fringes between the subcycle OV pulse and a fundamental subcycle pulse with a Gaussian mode. For all wavelength components, the root-mean-square (RMS) errors of the phases are <0.43$\pi$ rad, which is reasonably smaller than the 2$\pi$ rad of phases accumulating along a closed loop in the azimuth angle direction of the *ℓ*=1 OV pulse. The temporal profile is evaluated by adopting two-dimensional shearing interferometry (2DSI)^52^, and the resultant pulse duration is 4.7 fs, equivalent to 0.9 optical cycles at the carrier wavelength of 1.54 µm. We further utilize the acquired spectral and spatial amplitude/phase information to reconstruct three-dimensional (3D) isosurfaces representing the electric field and intensity profiles. Subsequently, we successfully control the rotation of the spiral interference fringes observed by the 2D $f$-2$f$ interferometry of the subcycle OV pulse upon the manipulation of the CEP. In Discussion, we summarize the results and describe the future prospects. Finally, in Materials and methods, we discuss the importance of the design for the beam splitter used in the SI.

## Results

### System overview

To generate and analyze subcycle OV pulses, we perform the following procedure steps: (1) Subcycle pulses are prepared with a TEM_00_ Gaussian mode, which involves an over-one-octave spectral component as a necessary condition. For simplicity, we omit “TEM_00_” hereafter. (2) The subcycle pulse is converted from the Gaussian mode to the Laguerre–Gaussian (LG) mode to form a subcycle OV pulse via a mode converter. (3) The temporal profile and the helical spatial wavefront of the converted OV pulse are characterized. (4) Finally, 2D $f$-$2f$ interferometry is performed to show the CEP control of the wavefront.

**Fig. 1:**
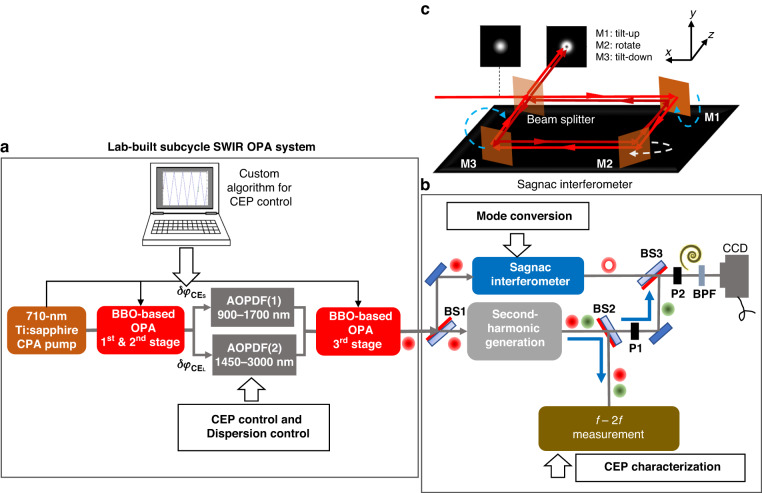
Schematic of the experimental setup for the generation and characterization of the subcycle OV pulse. **a** Lab-built OPA system to deliver subcycle pulses with a Gaussian mode to the mode converter. The CEP shift of the short-wavelength (0.9–1.45 µm) component $\delta {\varphi }_{{\rm{C}}{{\rm{E}}}_{{\rm{S}}}}$ and that of the long-wavelength (1.45–2.4 µm) component $\delta {\varphi }_{{\rm{C}}{{\rm{E}}}_{{\rm{L}}}}$ are synchronously controlled with AOPDF(1) and AOPDF(2) set between the second and third stages of the OPA chain, respectively. **b** Setup for performing conventional $f$-$f$ interferometry or 2D $f$-$2f$ interferometry. On **BS3**, the subcycle OV pulse with an LG mode coming from the Sagnac interferometer in the blue rectangle with round corners interferes with the reference fundamental or SH pulse with a Gaussian mode coming from the second-harmonic generator in a gray rectangle with round corners, and then the resultant beam profile is recorded with an IR CCD camera on the right panel of this figure. We can switch the reference pulse from the fundamental to the SH pulse by adjusting the rotation angle of the polarizer **P1** from 0° to 90°. The polarizer **P2** is used for adjusting the intensity ratio of the OV pulse to the reference SH pulse in the 2D $f$-$2f$ interferometry. The reference fundamental and SH pulses are partially sent to a conventional $f$-$2f$ interferometer in the brown rectangle with round corners to monitor the CEP shift. **BS1,** **BS2,** **BS3**: beam splitters. **P1,** **P2**: polarizers. CCD: IR CCD camera. **BPF**: bandpass filter. **c** Configuration of the Sagnac interferometer used as a mode converter. The reflective mirrors **M1,** **M2**, and **M3** are tilted to the directions indicated by the dashed arrows

A schematic of the experimental setup is shown in Fig. 1. In step (1) (Fig. 1a), we generate subcycle pulses with a Gaussian mode from a lab-built OPA system consisting of three stages of the OPA chain, in which a $\beta$-barium borate (BBO) crystal is used as a nonlinear gain medium^50,53^. A key feature to obtaining an over-one-octave bandwidth is the careful tuning of the pump wavelength in the OPA system to ~710 nm; this is the specific wavelength satisfying the broadband phase-matching condition in a BBO crystal to amplify the wavelength range from 0.9 µm to 2.4 µm. This wavelength range coincides with the ‘short-wave infrared (SWIR)’. Another key feature is an MZI inserted between the second and third stages of the OPA chain. This MZI splits the wavelength component into short- (0.9–1.45 µm) and long- (1.45–2.4 µm) wavelength components and combines them at its exit. By inserting an acousto-optic programmable dispersive filter (AOPDF, Dazzler:Fastlite) in each arm of the MZI to control the dispersion and the CEP of each wavelength component, we can obtain the entire range of the over-one-octave spectrum, even though the acceptable spectral width of each AOPDF is smaller than one octave. We denote the CEP of the short-wavelength component as ${\varphi }_{{\rm{C}}{{\rm{E}}}_{{\rm{S}}}}$ and that of the long-wavelength component as ${\varphi }_{{\rm{C}}{{\rm{E}}}_{{\rm{L}}}}$ following the notations used in ref. ^53^. The CEP shift given by each AOPDF is described as $\delta {\varphi }_{{\rm{C}}{{\rm{E}}}_{{\rm{J}}}}$, and the CEP is expressed as ${\varphi }_{{\rm{C}}{{\rm{E}}}_{{\rm{J}}}}=\delta {\varphi }_{{\rm{C}}{{\rm{E}}}_{{\rm{J}}}}+{\varphi }_{{\rm{C}}{{\rm{E}}}_{{{\rm{J}}}_{0}}}$ by using an unknown CEP offset ${\varphi }_{{\rm{C}}{{\rm{E}}}_{{{\rm{J}}}_{0}}}$, where ${\rm{J}}={\rm{S}},\,{\rm{L}}$. Note that the CEP of the synthesized pulse, ${\varphi }_{{\rm{CE}}}$, is approximately equal to $({\varphi }_{{\rm{C}}{{\rm{E}}}_{{\rm{S}}}}+{\varphi }_{{\rm{C}}{{\rm{E}}}_{{\rm{L}}}})/2$ when the field amplitude of the short-wavelength component is similar to that of the long-wavelength component and ${\varphi }_{{\rm{C}}{{\rm{E}}}_{{\rm{S}}}}-{\varphi }_{{\rm{C}}{{\rm{E}}}_{{\rm{L}}}}={\rm{const}}$. More details regarding the setup of the OPA system and the CEP control technique using the two AOPDFs can be found in refs. ^50^ and ^53^, respectively.

In step (2), we initially divide the generated Gaussian beam into two paths with a beam splitter, indicated as **BS1** in Fig. 1b. The pulse reflected from **BS1** is sent to an SI acting as a mode converter^44^. The schematic of the SI is depicted in Fig. 1c. The SI consists of three reflective mirrors (**M1,** **M2**, and **M3**) and a custom-coated $T/R$ = 50/50 beam splitter with a magnitude of group-delay-dispersion (GDD) of <10 fs^2^ in our target wavelength range (1.0–2.4 µm), where $T$ and $R$ are the transmittance and reflectance of the BS, respectively. We use the SI as the mode converter because any additional dispersion is reduced as low as possible if the beam splitter in the SI is carefully designed. In addition, the mode conversion using the SI is approximately achromatic over the entire wavelength range of the subcycle pulse, as shown in **Section S-2** **B** in the supplementary document. The OV pulse is generated by the interference from two collimated Gaussian beams, propagating in clockwise and counterclockwise directions in the SI with a relative shear displacement in the $y$ (vertical)-axis and a relative angular offset in the $x$ (horizontal)-axis. The shear displacement is implemented by vertically tilting **M1** and **M3** in opposite directions by an equal amount. We denote this displacement as ${d}_{y}$. The angular deviation is achieved by horizontally tilting **M2** around the $y$ axis. We denote the resultant angular offsets for the two counterpropagating Gaussian beams as ${\theta }_{x}$. The tilts of the three mirrors are finely controlled by kinematic mirror mounts with piezoelectric adjusters (Polaris, Thorlabs). An ideal OV beam at a particular wavelength ${\lambda }_{{\rm{r}}}$ is generated when the relationship ${kw}({\lambda }_{{\rm{r}}}){\theta }_{x}/2={d}_{y}/w({\lambda }_{{\rm{r}}})$ is satisfied, as explained in **Section S-2** **A** in the supplementary document, where $w({\lambda }_{{\rm{r}}})$ is the beam radius at ${\lambda }_{{\rm{r}}}$. We define ${\alpha }_{x}\equiv {kw}({\lambda }_{{\rm{r}}}){\theta }_{x}/2$ and ${\beta }_{y}\equiv {d}_{y}/w({\lambda }_{{\rm{r}}})$ to simplify the above relationship to ${\alpha }_{x}={\beta }_{y}$ in the following.

The temporal profile of the subcycle OV pulse is characterized by two-dimensional shearing interferometry (2DSI)^52^. The output pulse from the SI is reflected with a removable mirror (not shown in Fig. 1b) and directed to the instrument for the 2DSI. The details of this measurement are described in “Characterization of the temporal profile”.

The pulse transmitted through **BS1**, as shown in Fig. 1b, is sent to the SH generator consisting of a 1:1 telescope with a pair of focusing lenses and a nonlinear crystal placed at the focusing point of the telescope; this utilizes the generated SH pulse or the residual fundamental pulse with a Gaussian mode as a reference pulse in the $f$-$f$ or $f$-$2f$ interferometry in steps (3) or (4). We use a 3-mm-thick BBO crystal cut at ${\theta }_{{\rm{p}}}$ (polar angle) = 20° for the type-I phase-matching condition as the nonlinear crystal to generate the SH pulse. The fundamental ($f$-field) and SH ($2f$-field) pulses are partially reflected from **BS2**, as depicted in Fig. 1b, and directed to the conventional 1D $f$-$2f$ interferometer for the CEP measurement.

The SH and fundamental reference pulses with a Gaussian mode transmitted through **BS2** are sent to **BS3** in steps (3) and (4) to be superposed with the OV pulse sent from the SI. The beam divergence of the reference pulse is adjusted by using a movable lens, with a focal length of 100 mm, set behind **P1**, to show the spiral interference fringes with a suitable winding period. We can select only the fundamental pulse to be sent to **BS3** by setting the rotation angle of **P1** placed behind **BS2** in Fig. 1b to be 0° because the polarization direction of the fundamental pulse is parallel to the optical table. Conventional $f$-$f$ interferometry to evaluate the fidelity of the helical wavefront of the OV pulse can be performed with this setup. We can also select only the SH pulse by setting the rotation angle of **P1** to 90° because the polarization direction of the SH pulse is perpendicular to the optical table. 2D $f$-$2f$ interferometry can be performed with this configuration.

To perform the 2D $f$-$2f$ interferometry, the intensity ratio of the OV pulse to the SH reference pulse is adjusted to maximize the visibility of the spiral interference fringe by tuning the rotation angle of **P2** placed behind **BS3**. The beam profiles showing the interference fringes are recorded with an infrared (IR) CCD camera (Xeva-2.35, Xenics). Note that we can also record the OV pulse beam profiles by blocking the reference pulse beam path. We place a bandpass filter before the camera to detect the beam profile containing only the necessary wavelength component.

### Beam profile

**Fig. 2:**
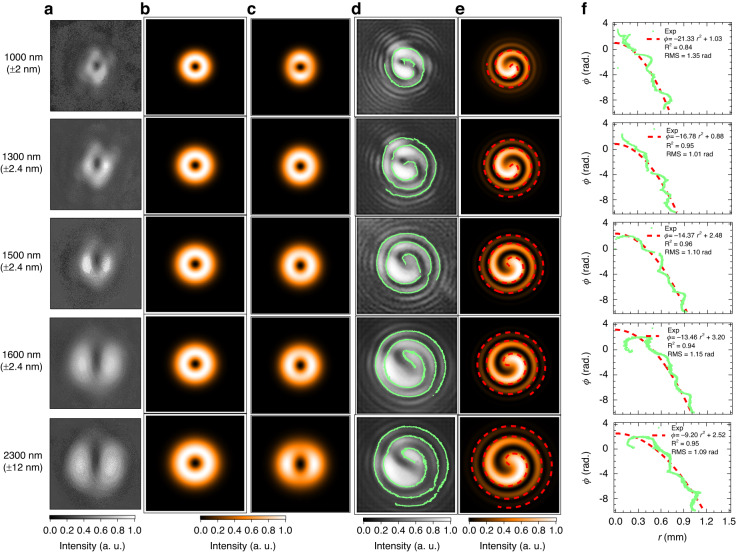
Characterization of the beam profiles and wavefronts. **a** Beam profiles of the output pulse from the SI recorded with an IR CCD camera set behind a bandpass filter transmitting in a wavelength range of 1000$\pm$2 nm (top panel), 1300$\pm$2.4 nm (second top panel), 1500$\pm$2.4 nm (center panel), 1600$\pm$2.4 nm (second bottom panel), or 2300$\pm$12 nm (bottom panel). **b** Beam profiles calculated using Eq. (S-13) in the supplementary document, expressing the exact LG mode. We assume that the beam waist is proportional to each wavelength shown on the left-hand side of each panel in (**a**). **c** Beam profiles calculated using Eq. (S-12) in the supplementary document, which includes the effect of chromatic aberration in the SI. **d** Beam profiles obtained from the experiment by $f$-$f$ interferometry, in which the output pulse from the SI is superposed on the fundamental reference pulse with a Gaussian mode. In each panel, the wavelength range is differentiated by the bandpass filter used to observe the beam profile shown in the panel in the same row in (**a**). The contour tracing one of the profile edges is depicted as the dotted curve in each panel. **e** Beam profiles calculated using Eq. (S-20) in the supplementary document to represent the $f$-$f$ interferometry. The contour tracing one of the profile edges is depicted as a dashed curve in each panel. **f** Contour in each panel of (**d**) (dotted curve) and that in each panel of (**e**) (dashed curve) on the plane of the cylindrical coordinate system. The latter curve is known as a Fermat’s spiral, and the azimuth angle $\phi$ is expressed as a quadratic function of the radius $r$

We show in the five panels of Fig. 2a the recorded images of the beam profiles of the subcycle OV pulse obtained by closing the beam path of the reference pulse. These panels are arranged in ascending order of the wavelength components separated by inserting a commercial bandpass filter transmitting in a wavelength range of 1000$\pm$2 nm, 1300$\pm$2.4 nm, 1500$\pm$2.4 nm, 1600$\pm$2.4 nm, or 2300$\pm$12 nm in front of the IR CCD camera. The beam profiles of the OV pulse calculated from the model equation of Eq. (S-13) in the supplementary document are depicted in Fig. 2b. All profiles exhibit doughnut-shaped structures with a homogeneous intensity distribution along the azimuth angle because the exact LG mode is assumed in these calculations. We also assume that the beam waist is proportional to the wavelength to mimic the trend of the monotonic increase in the beam waist of the experimental results in Fig. 2a upon the increase in the wavelength. However, this model of the exact LG mode does not reproduce the modulations of the intensity distribution along the azimuth angle observed in the experimental beam profiles in Fig. 2a. We discuss this issue in **Section S-2** **B** in the supplementary document and find that these modulations mainly come from the intrinsic chromatism originating from the SI. Once ${\alpha }_{x}={\beta }_{y}$ at a particular wavelength $\lambda ={\lambda }_{{\rm{r}}}$, for example, at 1500 nm, the beam profiles of the wavelength components other than 1500 nm become distorted with an increase in the wavelength difference from 1500 nm, as shown in Fig. 2c. The images in the five panels of this figure are calculated using Eq. (S-12) in the supplementary document, which includes the effect of chromatism in the SI. The beam profile is gradually stretched in the horizontal direction as the wavelength increases, and the intensity at the horizontal sides increases. In contrast, the intensity gradually decreases in the horizontal direction as the wavelength decreases and the intensity at the vertical sides increases. These features of the beam profiles are similar to those shown in Fig. 2a.

Even though the beam profiles are slightly modulated with a chromatic effect, the SI is a useful device as a mode converter because the mode purity of the simulated SI output is determined to be >95% in the entire wavelength range of the subcycle pulse, as described in **Section S-2** **B** in the supplementary document; this output is sufficiently high and provides an actual helical wavefront of an OV pulse.

### Characterization of wavefront

To confirm the phase distribution of the output pulse from the SI, we examine the $f$-$f$ interferometry of the output pulse from the SI by superposing the reference pulse with a Gaussian mode; this is the residual fundamental pulse from the SH generator in Fig. 1b. The fundamental pulse is chosen by fixing the rotation angle of **P1** to 0°. We show the resultant beam profiles for the spiral interference fringes at five wavelength components in Fig. 2d. In each panel of this figure, the wavelength component is discriminated by using the same bandpass filter as that used to observe the beam profile shown in the panel in the same row in Fig. 2a. The dotted curve in each panel is the contour indicating one of the edges of each spiral interference fringe extracted by the Canny edge detection method^54^. We also show the beam profile at each wavelength calculated using Eq. (S-20) in the supplementary document in each panel of Fig. 2e. This equation is derived by considering the following condition: an OV pulse with an LG mode, a wavefront curvature of ${R}_{{\rm{v}}}$ and topological charge of *ℓ*; this pulse is superposed with a reference pulse with a Gaussian mode, whose wavefront curvature is ${R}_{{\rm{g}}}$. To calculate each beam profile, the topological charge *ℓ* is fixed to $+1$, and other parameters are determined to mimic the experimental conditions. The contour tracing one of the edges of the spiral interference fringe is shown as a dashed curve in each panel.

The spiral structure of the interference fringe appearing in all the beam profiles in Fig. 2d shows the nature of the helical wavefront; thus, the output pulse from the SI is determined to be the OV pulse in the entire wavelength range. There is only 1 spiral; thus, we can determine the magnitude of the topological charge to be 1. These characteristics are confirmed by comparing the beam profiles obtained from the experiment (Fig. 2d) with those obtained from the calculation (Fig. 2e). This comparison can also determine the sign of the topological charge. From Eq. (S-20) in the supplementary material, the direction of the spiral winding around the center is reversed when the sign of the topological charge switches to the opposite. When the winding directions of the experimental and calculated spirals are both clockwise, the sign of the topological charge of the output pulse from the SI is positive, namely, *ℓ*$=+1$.

To identify the quality of the output pulse from the SI as an OV pulse, we evaluated the deviation and consistency of the wavefront by utilizing the spiral edge contour extracted from the image of the spiral interference fringe. The edge contours of the experimental results at five wavelength components are depicted as dotted curves on the $r$–$\phi$ plane of the cylindrical coordinate system in the panels of Fig. 2f. According to the analysis in **Section S-3** in the supplementary document, the edge contour should become a Fermat’s spiral curve, for which the azimuth angle $\phi$ is expressed as a quadratic function of radius $r$: $\phi =A{r}^{2}+B$. The coefficients $A$ and $B$ are determined by the beam parameters of the OV pulse and the reference pulse with a Gaussian mode. The analytical forms of $A$ and $B$ are provided in Eqs. (S-24) and (S-25), respectively, in the supplementary document. We fit the edge contours obtained from the experiment to the function $\phi =A{r}^{2}+B$ by adapting the coefficients $A$ and $B$ as fitting parameters. The resultant fits are shown as dashed curves in Fig. 2f.

We adopt the root mean square (RMS) error and R-squared value (R^2^) between the experimental edge contours and their fits to evaluate the deviation from the ideal OV pulse and the consistency of the ideal OV pulse, respectively. The resultant RMS error and R^2^ are listed in Table 1.

**Table 1:** RMS and R^2^ for evaluating wavefront

Wavelength (nm)	RMS error (rad)	R^2^
1000	1.35	0.84
1300	1.01	0.95
1500	1.10	0.96
1600	1.15	0.94
2300	1.09	0.95

From Table 1, the RMS error is 1.35 = 0.43*π* rad at most, which is sufficiently low to cause the $2\pi$-rad phase shift accumulated along a closed circle in the wavefront of the *ℓ* = 1 OV pulse. The low values of RMS errors are consistent with large R-squared values, which are evaluated to be ≥0.84, such that the experimental spiral contours highly correlate to the simulated contours following Fermat’s spirals, as shown in Fig. 2f.

As a summary of this section, we have generated the OV pulse with a topological charge of $+1$ in the entire wavelength range; the range is sufficiently broad such that the subcycle pulse can be formed with an adequate compensation of the spectral dispersion.

### Characterization of the temporal profile

To characterize the temporal profile of the generated OV pulse, we build a setup for implementing the 2DSI^52^ measurement just behind the SI. The OV pulse is sent to this setup with a removable mirror only when the 2DSI measurement is performed. In the 2DSI setup, a highly chirped pulse is generated by selecting a portion of the 710-nm pump pulse from the OPA system and dispersing it with two 14.3-mm-thick s-TIH6 (Ohara. Inc) glass plates under Brewster incidence. After the dispersion plates, the highly chirped pulse is split into two pulses in a Michelson interferometer, where a T/R = 50/50, 10-mm N-BK7 cube beam splitter is used. The two replica pulses are finally combined with the OV pulse in a type-II BBO crystal with a thickness of 0.1 mm for the sum frequency generation (SFG). The acceptable bandwidth in the SFG process is sufficiently large to convert the entire SWIR spectrum of the OV pulse to the visible region; this is measurable with a spectrometer equipped with an array of conventional silicon detectors. The setup resembles a typical 2DSI setup in^52^.

**Fig. 3:**
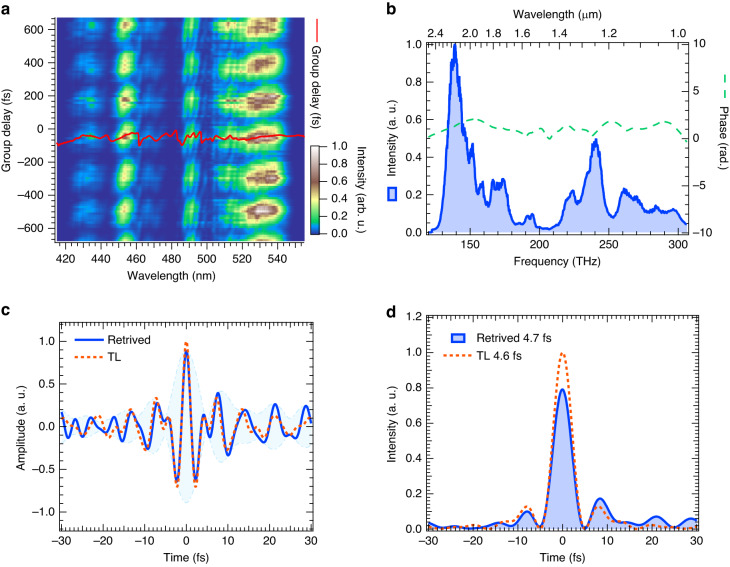
Characterization of the temporal profile through 2DSI. **a** 2DSI spectrogram of the OV pulse. The solid curve is the GD obtained from the spectrogram. **b** Measured spectrum (shaded area) of the OV pulse and the spectral phase (dashed line) retrieved from the measured GD in (**a**). **c** Electric field (solid curve) calculated from the measured spectrum and the retrieved phase. The shaded area is the envelope of the electric field. The dashed curve is the electric field of the TL pulse. The CEP is assumed to be $0$ to retrieve the electric fields. **d** Temporal profile (intensity) (shaded area) calculated from the measured spectrum and the retrieved phase compared with the TL pulse (dashed curve)

The spectrogram in Fig. 3a exhibits the spectrum sequence obtained while scanning the phase of one of the replica pulses in the pair. The phase for the vertical axis in this spectrogram is converted to the equivalent delay with a measured frequency shear of ~4.4 THz, determined by evaluating the frequency shift between the spectra of the two upconverted sheared pulses. The solid curve in Fig. 3a is the group delay (GD) of the measured pulse extracted from the spectrogram. The spectral phase is retrieved by integrating the GD for angular frequency. The spectral phase (dashed curve) and measured spectrum (solid curve with the shaded area) of the OV pulse are shown in Fig. 3b. Figure 3c depicts the retrieved electric field assuming zero CEP as a solid curve. The electric field of the transform-limit (TL) pulse is also shown as a dashed curve for comparison. From Fig. 3d, the pulse duration was determined to be 4.7 fs in full width at half-maximum (FWHM) from the temporal intensity profile (shaded curve) of the OV pulse retrieved from the spectral phase and spectrum. This was comparable to the duration of the TL pulse of 4.6 fs (dashed curve) in Fig. 3d. The pulse duration of 4.7 fs was equivalent to 0.9 optical cycles at the carrier wavelength of 1.54 µm, determined by the center-of-gravity frequency of the measured spectrum.

We deduce that the subcycle OV pulse is generated because of the spatial mode evaluation of an OV pulse in the entire spectral range described in “Beam profile” and “Characterization of wavefront” and the determination of the temporal profile described in this section. In this context, the term “subcycle pulse” designates a light or electromagnetic radiation pulse characterized by the FWHM of its intensity that is shorter than the oscillation cycle of the electric field at its central frequency. Specifically, the subcycle pulse represents an ultrashort optical burst lasting for a mere fraction of a single oscillation in the electric field within an optical cycle. Notably, despite its ultrashort duration, the time integral of the electric field for a subcycle pulse retains an exact and inherent average value of zero.

### Reconstruction of 3D electric field and intensity profiles

To reconstruct the time-dependent electric field $E(r,\phi ,{z}_{{\rm{F}}},t)$ of the OV pulses at a fixed propagation distance of $z={z}_{{\rm{F}}}$, we employ inverse Fourier transform techniques on $\widetilde{E}(r,\phi ,{z}_{{\rm{F}}},\omega )$ in the angular frequency domain. This transformation is carried out at each spatial point using Eq. (S-3), as outlined in the supplementary document.

For an ideal OV pulse, we assume a constant spectral phase for all wavelength components and a Fourier amplitude identical to that of an LG beam, as described in Eq. (S-2) in the supplementary document. The ideal spatial phase distribution ${\phi }_{{\rm{ideal}}}$ at each wavelength, represented by Fermat’s spirals, is provided in “Characterization of wavefront”.

In the case of the experimental OV pulse, we introduce a correction to the spatial phase distribution denoted as $\Delta \phi ={\phi }_{\exp }-{\phi }_{{\rm{ideal}}}$. Here, ${\phi }_{\exp }$ represents the spatial phase obtained from the experiment at various positions $r$ and angular frequencies $\omega$, as illustrated in Fig. 2f. Additionally, we incorporate the wavelength-dependent spectral phase retrieved in “Characterization of the temporal profile”, as shown in Fig. 3b. The Fourier amplitude, denoted as $\widetilde{\varepsilon }(r,\phi ,{z}_{{\rm{F}}},\omega )$, is determined by measuring the angularly resolved spectra $S(r,\phi ,{z}_{{\rm{F}}},\omega )$. This relationship is expressed as $\widetilde{\varepsilon }(r,\phi ,{z}_{{\rm{F}}},\omega )=\sqrt{S(r,\phi ,{z}_{{\rm{F}}},\omega )}$. To selectively transmit different transverse elements of the OV pulse, we use an aperture mounted on a translation stage. The emitted light is then collimated into a calibrated spectrometer (Ocean Optics, NIRQuest512) using a lens with a focal length of 4 cm, enabling the acquisition of angularly resolved spectra $S(r,\phi ,{z}_{{\rm{F}}},\omega )$. The intensity profile $I(r,\phi ,{z}_{{\rm{F}}},t)$ for the OV pulse is defined as ${\rm{|}}E(r,\phi ,{z}_{{\rm{F}}},t){{\rm{|}}}^{2}$. We assume that the phase error is homogeneously distributed along the azimuth direction except for the nonlinear phase error caused by the chromatic aberration in the SI described with Eq. (S-15) in the supplemental document. The characterization of the relative CEP is presented in the following section through the 2D $f$-2$f$ interferometry performed in the experiments, due to the stabilization and control of the CEP attained in our OPA system. Note that the absolute CEP was not determined in our measurement; thus, we assumed the absolute CEP to be 0 in the reconstruction of the electric field^55,56^.

**Fig. 4:**
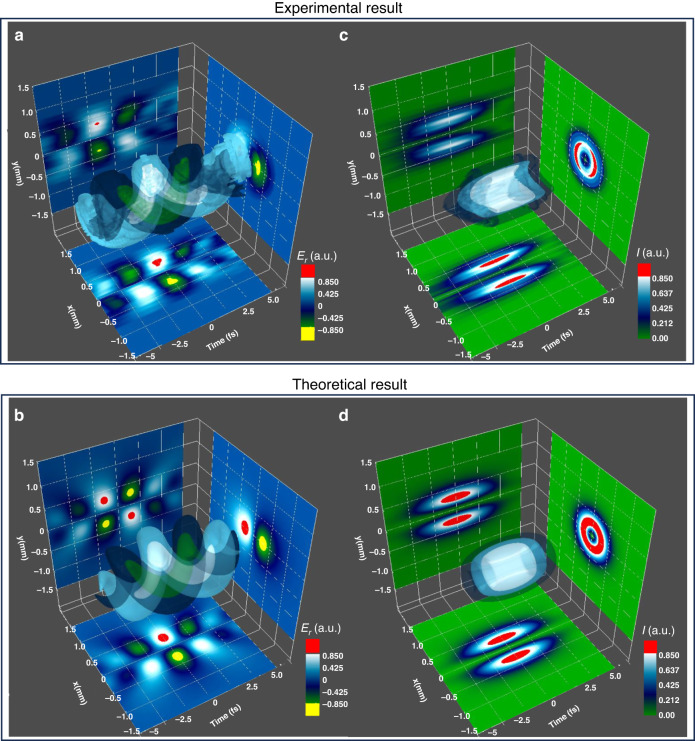
3D isosurfaces for the experimental and theoretical OV pulses. **a** 3D isosurface of the electric field of the subcycle OV pulse reconstructed from the measured data. Details of the reconstruction method are described in the main text. **b** 3D isosurface of the electric field of the subcycle OV pulse calculated from Eq. (S-3) in the supplementary document. **c** 3D isosurface of the intensity profile of subcycle OV shown in (**a**). **d** 3D isosurface of the intensity profile of subcycle OV shown in (**b**). Each isosurface is defined in a 3D space with the time of the moving frame ${t}_{{\rm{F}}}=t-{z}_{{\rm{F}}}/c$ at a fixed propagation coordinate $z={z}_{{\rm{F}}}$ and transverse spatial coordinates $x$ and $y$. The color code at the bottom right of each figure in (**a**, **b**) represents the amplitude of the electric field ${E}_{{\rm{r}}}(r,\phi ,{z}_{{\rm{F}}},t)$, with colors spanning values between $-1$ and 1 for the normalized field. Amplitudes below $-0.85$ and above 0.85 are highlighted in yellow and red, respectively. Figures (**a**, **b**) show electric fields ${E}_{{\rm{r}}}(r,\phi ,{z}_{{\rm{F}}},t)$ obtained by taking the real part of the complex field $E(r,\phi ,{z}_{{\rm{F}}},t)$. Figures (**c**, **d**) display spatiotemporal intensity profiles $I(r,\phi ,{z}_{{\rm{F}}},t)$ calculated as ${\rm{|}}E(r,\phi ,{z}_{{\rm{F}}},t){{\rm{|}}}^{2}$. The color code at the bottom right of these figures represents the intensity values, ranging from 0 to 1 for the normalized intensity, and the intensities exceeding 0.85 are highlighted in red

The 3D isosurface of the electric field of the OV pulse reconstructed with the abovementioned method is shown in Fig. 4a; this figure is compared with the 3D isosurface of the electric field of the ideal OV pulse simulated using Eq. (S-3) in the supplementary document with a pulse duration of 4.5 fs FWHM, as shown in Fig. 4b. The amplitudes of these fields are color-coded, with colors ranging from $-1$ to 1 to represent normalized field values. Yellow signifies amplitudes below $-0.85$, while red signifies amplitudes above 0.85. We also demonstrate a cross–sectional image of the isosurface at the ${t}_{{\rm{F}}}=0$ cut on the $x$–$y$ plane on the right-hand side in each figure and those at the $y=0$ and $x=0$ cuts on the $x$–${t}_{{\rm{F}}}$ and $y$–${t}_{{\rm{F}}}$ planes in a similar manner, where ${t}_{{\rm{F}}}\equiv t-{z}_{{\rm{F}}}/c$ specifies the time on the moving frame. Notably, the reconstructed electric field in Fig. 4a exhibits a similar spiraling pattern to that of the simulated electric field in Fig. 4b, serving as definite evidence of the successful generation of a subcycle OV pulse in our study. Figure 4c, d further show the 3D isosurfaces of the intensity counterparts and their cross-sectional images for Fig. 4a, b, respectively. These visual representations provide insights into the spatiotemporal characteristics of the electric fields and intensity profiles of the subcycle OV pulse. In **Section S-1** of the supplementary document, a more detailed discussion is provided regarding the manipulation of the CEP and its impact on the evolution of 3D isosurfaces of the electric fields and their intensity counterparts.

### Control of helical wavefront by CEP shift

We examined how the wavefront of the subcycle OV pulse changes upon scanning the CEP shift by switching the rotation angle of the polarizer **P1** from 0° to 90° to send the SH reference pulse in Gaussian mode to **BS3** in Fig. 1b. The over-one-octave spanning spectrum of the reference fundamental pulse and that of the subcycle OV pulse were both very broad, and the shortest-wavelength region of the OV pulse overlapped the longest-wavelength region of the SH reference pulse without the need for spectral broadening. This was one of the distinct features of the over-one-octave spanning subcycle pulse; here, the spectrum of the reference pulse needed to be broadened with self-phase modulation before the reference pulse was converted to the SH pulse if the reference and measured pulse were the conventional few-cycle pulses. As described in **Section S-4** in the supplementary document, the 2D $f$-2$f$ interferometry showed the rotation of the spatial interference fringes depending on the CEP. Notably, we could not observe these spatial interference fringes without the stabilization of the CEP in our OPA system. The rotation angle of the spiral interference fringe was governed by the offset of the azimuth angle or the coefficient $B$ of Fermat’s spiral described in Eq. (S-29) in the supplementary document; here, the CEP of the OV pulse ${\varphi }_{{\rm{C}}{{\rm{E}}}_{{\rm{v}}}}$ and that of the reference fundamental pulse ${\varphi }_{{\rm{C}}{{\rm{E}}}_{{\rm{g}}}}$ were used in the form of $({\varphi }_{{\rm{C}}{{\rm{E}}}_{{\rm{v}}}}-2{\varphi }_{{\rm{C}}{{\rm{E}}}_{{\rm{g}}}})=-{\varphi }_{{\rm{C}}{{\rm{E}}}_{{\rm{v}}}}+{\rm{const}}$. when the OV and reference fundamental pulse were generated from the same pulse. Specifically, the CEP of the OV pulse was coupled to the spatial wavefront and that its alteration could be detected by 2D $f$-$2f$ interferometry with the reference SH pulse with a Gaussian mode.

**Fig. 5:**
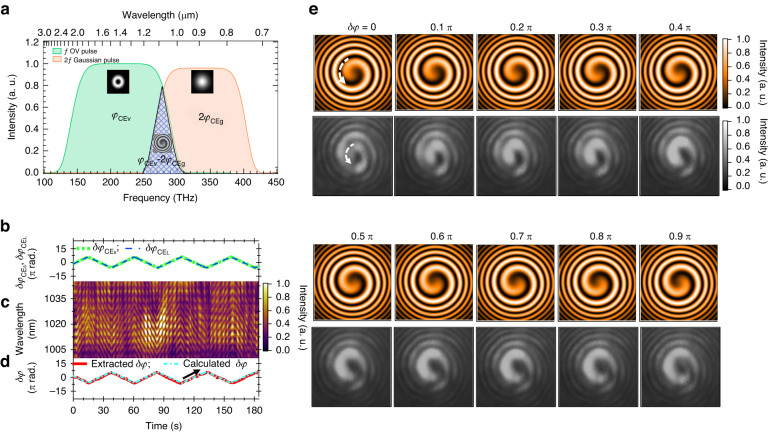
Control of the helical wavefront through CEP tailoring. **a** Schematic of the spectral interference between the fundamental ($f$) OV pulse with a CEP of ${\varphi }_{{\rm{C}}{{\rm{E}}}_{{\rm{v}}}}$ and the reference SH (2$f$) pulse with a Gaussian mode and a CEP of $2{\varphi }_{{\rm{C}}{{\rm{E}}}_{{\rm{g}}}}$. The overlap region interferes to generate a spiral fringe in the beam profile with an azimuth angle offset that includes the terms ${\varphi }_{{\rm{C}}{{\rm{E}}}_{{\rm{v}}}}-2{\varphi }_{{\rm{C}}{{\rm{E}}}_{{\rm{g}}}}$. **b** Evolution of the CEP shifts $\delta {\varphi }_{{\rm{C}}{{\rm{E}}}_{{\rm{S}}}}$ (dotted curve) and $\delta {\varphi }_{{\rm{C}}{{\rm{E}}}_{{\rm{L}}}}$ (dashed curve) applied to the two AOPDFs. **c** $f$-2$f$ Spectrogram measured using the reference fundamental and SH pulses in Gaussian mode. **d** Phase shift of the interference fringe $\delta \varphi$ extracted from the measured spectrogram in (**c**) (solid curve) and the CEP shift $\delta \varphi$ calculated from $\delta {\varphi }_{{\rm{C}}{{\rm{E}}}_{{\rm{S}}}}-2\delta {\varphi }_{{\rm{C}}{{\rm{E}}}_{{\rm{L}}}}$ (dashed-dotted curve). **e** Evolution of the spiral interference fringe upon the increase in $\delta \varphi$. The beam profiles in the upper rows are the simulated results obtained using Eq. (S-27) in the supplementary document. The beam profiles in the lower rows are the experimental results obtained using the IR CCD camera

Moreover, the CEP shift of the short-wavelength region $\delta {\varphi }_{{\rm{C}}{{\rm{E}}}_{{\rm{S}}}}$ and that of the long-wavelength region $\delta {\varphi }_{{\rm{C}}{{\rm{E}}}_{{\rm{L}}}}$ were synchronously controlled by AOPDF(1) and AOPDF(2), respectively, as shown in Fig. 1a. This experimental condition was already described in “System overview”. Based on the overlap wavelength region between the fundamental pulse and its SH pulse, the CEP shift of the OV pulse $\delta {\varphi }_{{\rm{C}}{{\rm{E}}}_{{\rm{v}}}}$ was the same as $\delta {\varphi }_{{\rm{C}}{{\rm{E}}}_{{\rm{S}}}}$ and the CEP shift of the reference SH pulse $2\delta {\varphi }_{{\rm{C}}{{\rm{E}}}_{{\rm{g}}}}$ was the same as $2\delta {\varphi }_{{\rm{C}}{{\rm{E}}}_{{\rm{L}}}}$; thus, the offset of the azimuth angle included the term $\delta {\varphi }_{{\rm{C}}{{\rm{E}}}_{{\rm{S}}}}-2\delta {\varphi }_{{\rm{C}}{{\rm{E}}}_{{\rm{L}}}}$, as described in Eq. (S-29) in the supplementary document. This situation is schematically shown in Fig. 5a. The analytical form describing the beam profile emerging in the 2D $f$-$2f$ interferometry is given by Eq. (S-27) in the supplementary document.

In the experiment, we also used a conventional 1D $f$-2$f$ interferometer, as shown in Fig. 1b, to record the spectrograms exhibiting interference between the reference fundamental ($f$) and the SH (2$f$) pulses with a Gaussian mode. The evolution of the spiral interference fringes was simultaneously obtained by using an IR CCD camera upon synchronously scanning $\delta {\varphi }_{{\rm{C}}{{\rm{E}}}_{{\rm{S}}}}$ and $\delta {\varphi }_{{\rm{C}}{{\rm{E}}}_{{\rm{L}}}}$.

We first examined whether the 1D $f$-2$f$ spectrogram could follow the evolution of the CEP shifts by synchronously applying triangular scanning waveforms of $\delta {\varphi }_{{\rm{C}}{{\rm{E}}}_{{\rm{S}}}}$ and $\delta {\varphi }_{{\rm{C}}{{\rm{E}}}_{{\rm{L}}}}$, as shown in Fig. 5b. We applied the same scanning waveforms of $\delta {\varphi }_{{\rm{C}}{{\rm{E}}}_{{\rm{S}}}}$ and $\delta {\varphi }_{{\rm{C}}{{\rm{E}}}_{{\rm{L}}}}$ to maintain the relationship of $\delta {\varphi }_{{\rm{C}}{{\rm{E}}}_{{\rm{S}}}}=\delta {\varphi }_{{\rm{C}}{{\rm{E}}}_{{\rm{L}}}}$ or ${\varphi }_{{\rm{C}}{{\rm{E}}}_{{\rm{S}}}}-{\varphi }_{{\rm{C}}{{\rm{E}}}_{{\rm{L}}}}={\rm{const}}$ during the scanning. The data acquisition conditions and the parameters used for this CEP scanning are listed in the first row of Table S-2 in the supplementary document.

We observed that the $f$-$2f$ interference fringes evolved with a triangular waveform in the spectrogram shown in Fig. 5c. Moreover, the waveform of the phase modulation extracted from the spectrogram $\delta \varphi$ depicted as solid lines in Fig. 5d became nearly the same as the waveform of $\delta {\varphi }_{{\rm{C}}{{\rm{E}}}_{{\rm{S}}}}-2\delta {\varphi }_{{\rm{C}}{{\rm{E}}}_{{\rm{L}}}}$ depicted as dot-dashed lines; this result was expected from the principle of $f$-2$f$ interferometry with the adjustment of the offset of the extracted phase.

Next, 2D $f$-$2f$ interferometry was performed upon scanning $\delta {\varphi }_{{\rm{C}}{{\rm{E}}}_{{\rm{S}}}}$ and $\delta {\varphi }_{{\rm{C}}{{\rm{E}}}_{{\rm{L}}}}$ with the waveforms, as shown in Fig. 5b. We simultaneously recorded a sequence of images exhibiting the spiral interference fringe and a sequence of spectral traces exhibiting the $f$-$2f$ interference fringes to confirm that the relationship of $\delta \varphi =\delta {\varphi }_{{\rm{C}}{{\rm{E}}}_{{\rm{S}}}}-2\delta {\varphi }_{{\rm{C}}{{\rm{E}}}_{{\rm{L}}}}$ was preserved during scanning. We selected the images of the spiral interference fringe at every $0.1\pi$ increment in $\delta \varphi$ from the recorded image sequence, and these are shown in the bottom panels in Fig. 5e. A real-time movie with a rate of 30 frames per second for the recorded image sequence is provided as Video 1 in the supplementary material. We also showed the beam profile calculated from Eq. (S-27) in the supplementary document above each panel of the recorded beam profile at the same value of $\delta \varphi$ for comparison. As shown in the central region of the spiral fringe in each recorded beam profile, the fringe rotated in a counterclockwise direction upon the increase in $\delta \varphi$, similar to the calculated fringe; this direction is denoted by the dashed arrows in the leftmost top and bottom panels in Fig. 5e. The magnitude of the rotation angle was also the same as that of $\delta \varphi$. Therefore, the CEP of the OV pulse in our experiment was successfully controlled.

Owing to the separate control of $\delta {\varphi }_{{\rm{C}}{{\rm{E}}}_{{\rm{S}}}}$ and $\delta {\varphi }_{{\rm{C}}{{\rm{E}}}_{{\rm{L}}}}$, we performed another type of CEP scanning, during which the relationship of $\delta {\varphi }_{{\rm{C}}{{\rm{E}}}_{{\rm{S}}}}+\delta {\varphi }_{{\rm{C}}{{\rm{E}}}_{{\rm{L}}}}=0$ or ${\varphi }_{{\rm{C}}{{\rm{E}}}_{{\rm{S}}}}+{\varphi }_{{\rm{C}}{{\rm{E}}}_{{\rm{L}}}}={\rm{const}}$. was maintained, as demonstrated in Video 2 in the supplementary material. This CEP scanning could strongly modulate the pulse envelope; thus, we could not approximate the CEP of the synthesized pulse to be $({\varphi }_{{\rm{C}}{{\rm{E}}}_{{\rm{S}}}}+{\varphi }_{{\rm{C}}{{\rm{E}}}_{{\rm{L}}}})/2$. This complexity was intrinsic to synthesizing two different wavelength components, and we discussed the spatiotemporal characteristics specific to this synthesis in **Section S-1** in the supplementary document.

## Discussion

In this study, we generated a subcycle OV pulse by adopting the SI as a mode converter from a Gaussian mode to an LG mode with an orbital angular momentum *ℓ* of 1; here, the subcycle pulse with a Gaussian mode was delivered from the lab-built OPA system^50^ equipped with CEP controllers in two separate wavelength components^53^. With the quasi-achromatic property of the SI, the OV pulse was achieved in the entire wavelength range in the output pulse from the SI. This result was confirmed by evaluating the helical wavefronts via the $f$-$f$ interferometry between the OV pulse and the reference fundamental pulse with a Gaussian mode at multiple wavelength components selected to represent most of the entire spectral range. The RMS errors of the phase distribution contours extracted from the measured interference images were all ≤0.43$\pi$ rad; their RMS values were sufficiently smaller than the 2$\pi$ rad from the phase accumulation of the helical wavefront in one optical cycle of the *ℓ*=1 OV pulse. We utilized Fermat’s spiral curves to extract the phase distribution contours from the spiral interference fringes.

The temporal profile of the OV pulse was characterized by the 2DSI method. As a result, the pulse duration was estimated to be 4.7 fs, equivalent to 0.9 optical cycles at the carrier wavelength of 1.54 µm. Because of the helical wavefront and the subcycle pulse duration, the generation of the subcycle OV pulse in the SWIR region via our present method was successful. We also performed the reconstruction of 3D isosurfaces for the experimental OV pulses, utilizing the measured phase information and amplitudes under the assumption that the absolute CEP was 0. The reconstructed electric field of the OV pulse showed a strikingly similar spiraling structure to that of the ideal subcycle OV pulse and provided visual support for the spatiotemporal features of a subcycle OV pulse generated in our experiment.

The determination of the absolute CEP is necessary for the complete reconstruction of the electric field. To achieve this goal, we demonstrated the relative CEP control of the subcycle OV pulse by modulating the CEP shifts ($\delta {\varphi }_{{\rm{C}}{{\rm{E}}}_{{\rm{S}}}}$, $\delta {\varphi }_{{\rm{C}}{{\rm{E}}}_{{\rm{L}}}}$) for the two wavelength components with the two AOPDFs in our CEP-stabilized OPA system. To evaluate CEP controllability, we newly developed a 2D $f$-$2f$ interferometer, in which the fundamental OV pulse interfered with the reference SH pulse in Gaussian mode. We successfully observed the rotation of the spiral interference fringe in the beam profile upon changing $\delta {\varphi }_{{\rm{C}}{{\rm{E}}}_{{\rm{S}}}}-2\delta {\varphi }_{{\rm{C}}{{\rm{E}}}_{{\rm{L}}}}$. This result provided clear evidence that the CEP was coupled to the spatial wavefront in the OV pulse, and the 2D $f$-$2f$ interferometry enabled the characterization of the CEP control of an OV pulse by directly observing the rotation of the spiral interference fringe. The successful preparation of the CEP controllable OV pulses was the first step to measure the absolute CEP of the OV pulse, and our study could facilitate new research needing this type of spatiotemporal light source for applications in light–matter interactions.

## Materials and methods

The $T$ and $R$ of the beam splitter in the SI needs to be completely the same in the entire wavelength range to exactly locate the phase singular point in the center of the beam profile. In **Section S-2** **C** in the supplementary document, a theoretical model of the mode conversion is described and the effect on the parameters ${d}_{y}$, ${\theta }_{x}$, $T$, and $R$ for the generation of the OV pulse is discussed. In our experiment, the beam splitter is specially designed to let the ratio $T/R$ be ${1}_{-0.05}^{+0.1}$ in the entire wavelength range. The optical path length in the substrate material (CaF_2_) of the BS for the pulse propagating clockwise is the same as that for the pulse propagating counterclockwise. Thus, the dispersions of the BS substrate accumulated in both pulses are the same. The throughput efficiency of the SI is ~30%, which indicates an output energy of ~9.6 µJ with an input energy of 32 µJ.

### Supplementary information

Supplementary material

Video 1

Video 2

Video 3

## Data Availability

Data supporting the results presented in this paper are not publicly available at this time but may be obtained from the authors upon reasonable request.
