# Supplementary material for: Wavefront control of subcycle vortex pulses via carrier-envelope-phase tailoring
Source: Light Sci Appl. 2023 Nov 24;12:279. doi: 10.1038/s41377-023-01328-7 (PMC10667496; doi:10.1038/s41377-023-01328-7)
Supplement: Supplementary file 1 — Supplementary material [file 41377_2023_1328_MOESM1_ESM.pdf]

Supplementary information for: Wavefront control of subcycle vortex pulses via carrier-envelope-phase tailoring

Yu-Chieh Lin,* Katsumi Midorikawa, and Yasuo Nabekawa
Attosecond Science Research Team, RIKEN Center for Advanced Photonics, 2-1 Hirosawa, Wako, Saitama 351-0198, Japan

Abstract

This document provides supplementary information for "Wavefront control of subcycle vortex pulses via carrier-envelope-phase tailoring". We show the analysis of the waveform of the subcycle optical vortex (OV) pulse upon changing the carrier-envelope phase (CEP) of the short-wavelength spectral component $\varphi_{\mathrm{CE_S}}$ and that of the long-wavelength spectral component $\varphi_{\mathrm{CE_L}}$. We also present analytical formulae showing how a Sagnac interferometer (SI) converts a Gaussian beam to an OV pulse and then explain the characteristics of the spiral interference fringes appearing in the 2D f-f and f-$2f$ interferometries between the OV pulse and the Gaussian beam.

S-1. ANALYSIS OF SPATIOTEMPORAL CHARACTERISTICS OF SYNTHESIZED SUBCYCLE OV PULSES

A. Formulation

Owing to the division and synthesis of the wavelength region of the subcycle OV pulse as described in the main text, we use two parameters, $\delta\varphi_{\mathrm{CE_S}}$ and $\delta\varphi_{\mathrm{CE_L}}$, to control the CEP in our experiment. In this section, we show how these parameters alter the spatiotemporal form of the electric field of the OV pulse under the constraints of $\delta\varphi_{\mathrm{CE_S}} \pm \delta\varphi_{\mathrm{CE_L}} = 0$, as applied in our experiment. According to the basic theory of the OV field [1], we employ the expression of a Laguerre–Gaussian (LG) beam with a linear polarized amplitude whose spatial mode in cylindrical coordinates is proportional to the LG mode function $\mathrm{LG}_p^{\ell}(r,\phi,z)$, which is defined as

$$\mathrm{LG}_p^{\ell}(r,\phi,z) = \tilde{u}_p^{\ell}(r,\phi,z)\mathrm{e}^{-ikz-ik\frac{r^2}{2R(z)}-i\Theta_p^{\ell}(z)-i\ell\phi}, \tag{S-1}$$

where the real amplitude $\tilde{u}_p^{\ell}(r,\phi,z)$ is given by

$$\tilde{u}_p^{\ell}(r,\phi,z) = \sqrt{\frac{2p!}{\pi(p+|\ell|)!}}\frac{1}{w(z)}\left(\frac{\sqrt{2}r}{w(z)}\right)^{|\ell|} L_p^{|\ell|}\left(\frac{2r^2}{w^2(z)}\right)\exp\left(-\frac{r^2}{w^2(z)}\right), \tag{S-2}$$

where w_0 is the beam waist at focus, $w(z) = w_0\sqrt{1+(z/z_{\mathrm{R}})^2}$ is the beam radius, and $z_{\mathrm{R}} = \pi w_0^2/\lambda = kw_0^2/2$ is the Rayleigh length associated with the wavelength λ or the wavenumber $k = \omega/c$. We define the angular frequency as ω and the velocity of light in vacuum as c. $\mathrm{L}_p^{|\ell|}(x)$ is the associated Laguerre polynomial of the order p and degree $|\ell|$ with a variable x. $R(z) = z[1+(z_{\mathrm{R}}/z)^2]$ is the wavefront radius of curvature. $\Theta_p^{\ell}(z) = -(2p+|\ell|+1)\tan^{-1}(z/z_{\mathrm{R}})$ is the Gouy phase. The radial distance r and azimuth angle ϕ are given by $r = \sqrt{x^2+y^2}$ and $\phi = \tan^{-1}(y/x)$, respectively. The index ℓ, whose magnitude is included in the degree of the associated Laguerre polynomial, takes integers ($\ell = 0, \pm1, \pm2, ...$), and it physically corresponds to the topological charge of the helical wavefront. The order p takes a non-negative integer ($p = 0, 1, 2...$) and it gives the number of radial nodes of the OV field. The LG mode function is normalized so that the relation $\int_0^{\infty}drr\int_0^{2\pi}d\phi|\mathrm{LG}_p^{\ell}(r,\phi,z)|^2 = \int_0^{\infty}drr\int_0^{2\pi}d\phi|\tilde{u}_p^{\ell}(r,\phi,z)|^2 = 1$ should be satisfied.

* yu-chieh.lin@riken.jp

To express the CEP, we introduce the Fourier amplitude of the temporal envelope as $\tilde{\varepsilon}_c(\omega-\omega_c)\mathrm{e}^{i\varphi_{\mathrm{CE}_c}}$, where $\tilde{\varepsilon}_c(\omega)$ expresses an amplitude in the angular frequency domain that exhibits a magnitude peak at around $\omega=0$, ω_c is the carrier angular frequency, and φ_{CE_c} is the CEP with a carrier angular frequency of ω_c. The electric field of the OV pulse $E_p^\ell(r,\phi,z,t)$ is obtained by the inverse Fourier transform of $\tilde{\varepsilon}_c(\omega-\omega_c)\mathrm{e}^{i\varphi_{\mathrm{CE}_c}}\mathrm{LG}_p^\ell(r,\phi,z)$. Nevertheless, it is not easy to perform this transform because $\mathrm{LG}_p^\ell(r,\phi,z)$ implicitly depends on ω through the dispersion relation $k=\omega/c$. Thus, we approximate the inverse Fourier transform, as follows:

$$\begin{aligned}E_p^\ell(r,\phi,z,t) &= \int d\omega\tilde{\varepsilon}_c(\omega-\omega_c)\tilde{u}_p^\ell(r,\phi,z)\mathrm{e}^{-ik\frac{r^2}{2R(z)}-i\Theta_p^\ell(z)-i\ell\phi}\mathrm{e}^{i\omega(t-\frac{z}{c})}\mathrm{e}^{i\varphi_{\mathrm{CE}_c}}\\ &\simeq \widetilde{u_c}_p^\ell(r,\phi,z)\mathrm{e}^{-ik_c\frac{r^2}{2R_c(z)}-i\Theta_{p,c}^\ell(z)-i\ell\phi}\varepsilon_c(t-\frac{z}{c})\mathrm{e}^{i\omega_c t-ik_c z}\mathrm{e}^{i\varphi_{\mathrm{CE}_c}},\end{aligned} \tag{S-3}$$

in which we neglect the ω dependence of $\tilde{u}_p^\ell(r,\phi,z)$ and the phase term of the first exponent, and we replace ω with ω_c. The subscript $_c$ in these quantities on the right-hand side of the second line in Eq. (S-3) denotes this substitution.

To further simplify the analysis in the following, we only consider the temporal characteristic of the transverse mode at the propagation distance of $z=z_\mathrm{F}$, which is sufficiently far to apply the approximations of $1/R_c(z_\mathrm{F})\sim 0$ and $\Theta_{p,c}^\ell(z_\mathrm{F})\sim -(2p+|\ell|+1)\pi/2$. We also restrict ourselves to adopting $p=0$, $\ell=\pm1$, and $\varepsilon_c(t)$ is a real function expressing the Fourier-transform-limit pulse envelope. To reproduce the experimental condition under which a few-cycle pulse composed of the short-wavelength component is superposed with another few-cycle pulse composed of the long-wavelength component, we assume that both few-cycle pulses can be expressed using the approximated Eq. (S-3) with the carrier angular frequencies of ω_S and ω_L. Thus, the electric fields of these two few-cycle OV pulses can be expressed as

$$E_\mathrm{J}^\ell(r,\phi,z_\mathrm{F},t) = -\,\varepsilon_\mathrm{J}(t_\mathrm{F})\frac{2}{\sqrt{\pi}}\frac{r}{w_\mathrm{J}^2}\,\mathrm{e}^{-\frac{r^2}{w_\mathrm{J}^2}}\mathrm{e}^{i(\omega_\mathrm{J}t_\mathrm{F}+\varphi_{\mathrm{CE}_\mathrm{J}}-\ell\phi)}, \tag{S-4}$$

where we define the time on the moving frame as $t_\mathrm{F}\equiv t-z_\mathrm{F}/c$. The minus sign before $\varepsilon_\mathrm{J}(t_\mathrm{F})$ comes from the Gouy phase $\mathrm{e}^{-i\Theta_{p,c}^\ell(z_\mathrm{F})}=\mathrm{e}^{i\pi}=-1$ in the far field. The subscript $\mathrm{J}\in\{\mathrm{S},\mathrm{L}\}$ represents the contributions from the short- and long-wavelength components. The notation of the beam radius at z_F is reduced from $w_\mathrm{J}(z_\mathrm{F})$ to w_J. We have used the relation $L_0^\ell(x)=1$ for the associated Laguerre polynomial and omitted the constant phase factors.

We further assume that the temporal envelopes of both wavelength components are the same for simplicity, i.e., $\varepsilon_\mathrm{S}(t)=\varepsilon_\mathrm{L}(t)\equiv\overline{\varepsilon}(t)$. In addition, we replace the beam radii of w_S and w_L with the common averaged beam radius of $\overline{w}=(w_\mathrm{S}+w_\mathrm{L})/2$ to roughly estimate the CEP evolution on the synthesized waveform $E_\mathrm{syn}^\ell(r,\phi,z_\mathrm{F},t)\equiv[E_\mathrm{S}^\ell(r,\phi,z_\mathrm{F},t)+E_\mathrm{L}^\ell(r,\phi,z_\mathrm{F},t)+\mathrm{c.c.}]/\sqrt{2}$, which can be expressed as

$$E_\mathrm{syn}^\ell(r,\phi,z_\mathrm{F},t)\approx-\overline{\varepsilon}(t_\mathrm{F})\sqrt{\frac{8}{\pi}}\frac{r}{\overline{w}^2}\,\mathrm{e}^{-\frac{r^2}{\overline{w}^2}}\cos\left[(\Delta\omega t_\mathrm{F}+\Delta\varphi_\mathrm{CE})/2\right]\cos\left(\overline{\omega}t_\mathrm{F}+\overline{\varphi_\mathrm{CE}}-\ell\phi\right), \tag{S-5}$$

where $\Delta\omega=\omega_\mathrm{S}-\omega_\mathrm{L}$, $\Delta\varphi_\mathrm{CE}=\varphi_{\mathrm{CE}_\mathrm{S}}-\varphi_{\mathrm{CE}_\mathrm{L}}$, $\overline{\omega}=(\omega_\mathrm{S}+\omega_\mathrm{L})/2$, and $\overline{\varphi_\mathrm{CE}}=(\varphi_{\mathrm{CE}_\mathrm{S}}+\varphi_{\mathrm{CE}_\mathrm{L}})/2$. The CEP of the synthesized field, $\varphi_{\mathrm{CE}_\mathrm{syn}}$, equals the average of the CEPs of the

two wavelength components, $\overline{\varphi_{\rm CE}}$, when $\Delta\varphi_{\rm CE} = 0$, because the synthesized field envelope modulates with a function of $\cos[(\Delta\omega t + \Delta\varphi_{\rm CE})/2]$. Furthermore, the intensity envelope of the synthesized waveform can be obtained from the square of the leading term before $\cos(\overline{\omega_{\rm c}}t + \overline{\varphi_{\rm CE}} - \phi)$, namely,

$$I^{\ell}_{\rm syn}(r,\phi,z_{\rm F},t) = \bar{\varepsilon}^2(t_{\rm F})\frac{4}{\pi}\frac{r^2}{\overline{w}^4}\exp\left(-\frac{2r^2}{\overline{w}^2}\right)[1+\cos(\Delta\omega t_{\rm F} + \Delta\varphi_{\rm CE})], \tag{S-6}$$

which is independent of ℓ. We utilize Eqs. (S-5) and (S-6) for the calculations in **Sec. S-1 B**.

B. Calculation results

In the experiment, we adopted two types of combination of CEP scanning routes [2] for $(\varphi_{\rm CE_S}, \varphi_{\rm CE_L})$ expressed as $\varphi_{\rm CE_S} \pm \varphi_{\rm CE_L} = $ const., and the constant value on the right-hand side of this equation was unknown. Nevertheless, we set this value to 0 in the following analysis to provide typical examples of modulations of the synthesized waveform with CEP control. We show the spatiotemporal evolution of the synthesized subcycle OV pulse along these two scanning routes calculated using Eqs. (S-5) and (S-6).

The carrier wavelengths used for the calculation are given as 1.2 μm and 1.9 μm for the short- and long-wavelength components, which correspond to the angular frequencies of $\omega_{\rm S} \approx 1571$ THz and $\omega_{\rm L} \approx 992$ THz, respectively. We have chosen the topological charge ℓ to be +1. Figures **S-1**(a)–(c) schematically demonstrate the three-dimensional (3D) isosurfaces of the electric field of the subcycle OV pulse $E_{\rm syn}$ in Eq. (S-5) at different CEP pairs of $(\varphi_{\rm CE_S}, \varphi_{\rm CE_L}) = (0,0)$, $(\pi/4\ ,\ \pi/4)$, and $(\pi/2\ ,\ \pi/2)$, respectively, on the scanning route of $\varphi_{\rm CE_S} - \varphi_{\rm CE_L} = 0$. These isosurfaces are transversely and temporally restricted by the envelope function in front of $\cos(\overline{\omega}t_{\rm F} + \overline{\varphi_{\rm CE}} - \phi)$ in Eq. (S-5). The amplitude of the field is indicated by a color code shown at the bottom right of Fig. **S-1**(c), where colors are set between −1 and 1 values of the normalized field. The amplitudes less than −0.85 and larger than 0.85 are highlighted in yellow and red, respectively. We also show a cross-sectional image of the isosurface at the $t_{\rm F} = 0$ cut on the x–y plane on the right-hand side in each figure, and those at the $y = 0$ and $x = 0$ cuts on the x–$t_{\rm F}$ and y–$t_{\rm F}$ planes in a similar manner. We observe that the spiraling nature of the wavefront and the shape of the field envelope are common in Figs. **S-1**(a)–(c), whereas the positions of the (positive) maximum and (negative) minimum of the field amplitude at the $t_{\rm F} = 0$ cut rotate clockwise with the amount of $\varphi_{\rm CE_S}(= \varphi_{\rm CE_L})$, as shown on the x–y plane with a dashed arrow in each figure. The rotation of the wavefront is also confirmed by the cross-sectional images on the x–$t_{\rm F}$ and y–$t_{\rm F}$ planes in which the maxima and minima of the field amplitudes move to the negative direction of time with an increment in the 1/8 period of carrier frequency. We note that the CEPs of all the electric fields in Figs. **S-1**(a)–(c) evolve with the same amount of the azimuth angle and with different CEP offsets. This is the intrinsic nature of the OV pulse with an $\ell = 1$ wavefront. Figures **S-1**(d)–(f) exhibit the isosurfaces of the 3D intensity spatiotemporal profile $I_{\rm syn}$ and their cross-sectional images common to all the three fields in Figs. **S-1**(a)–(c), where the zero intensity center at the vortex axis is observed.

This is in contrast to the modulation of the intensity envelope $I_{\rm syn}$ in the case of CEP scanning along a different route of $\varphi_{\rm CE_S} + \varphi_{\rm CE_L} = 0$, as shown in Fig. **S-2**. Figures **S-2**(a)–(c) depict the isosurfaces for the electric field $E_{\rm syn}$ at $(\varphi_{\rm CE_S}, \varphi_{\rm CE_L}) = (0,0)$, $(-\pi/4\ ,\ \pi/4)$, and

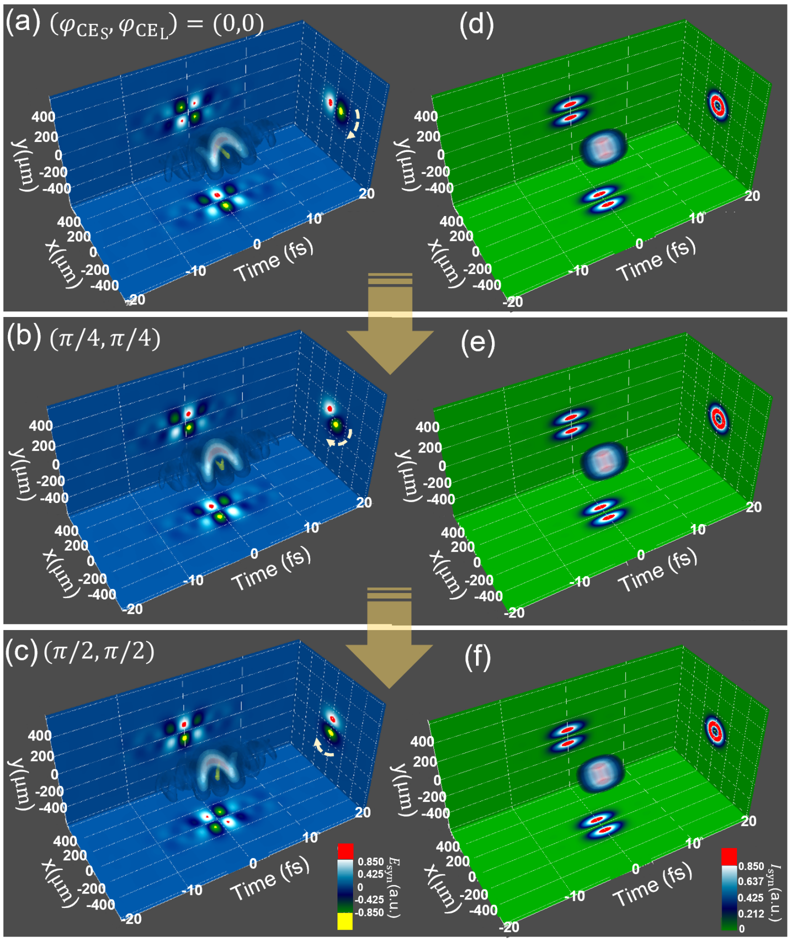

Fig. S-1. Isosurfaces of the electric field E_{syn} (a) with a CEP pair $(\varphi_{\mathrm{CE_S}}, \varphi_{\mathrm{CE_L}})$ of $(0, 0)$, (b) with a CEP pair of $(\pi/4, \pi/4)$, and (c) with a CEP pair of $(\pi/2, \pi/2)$. All the CEP pairs adopted in these calculations are on the scanning route satisfying $\varphi_{\mathrm{CE_S}} - \varphi_{\mathrm{CE_L}}$=0. Each isosurface is expressed by using the 3D variables of the time of the moving frame t_{F} and the transverse spatial coordinates x and y. The amplitude of the field is indicated by a color code depicted at the bottom right of Fig. **S-1**(c), where colors are set between -1 and 1 values of the normalized field. The amplitudes less than -0.85 and larger than 0.85 are highlighted in yellow and red, respectively. The dashed arrow on the x–y plane specifies the rotational direction of the wavefront at the cut of $t_{\mathrm{F}} = 0$ with the evolution of CEPs. (d)–(f) Isosurfaces of the spatiotemporal intensity profile I_{syn} obtained using Eq. (S-6). The values of the CEP pair used for the calculations to obtain **S-1**(d)–(f) are the same as those shown at the top left in Figs. **S-1**(a)–(c), respectively. The intensity is indicated by a color code at the bottom right of Fig. **S-1**(f), where colors are set between 0 and 1 values of the normalized intensity. Intensities larger than 0.85 are highlighted in red.

$(-\pi/2\ ,\ \pi/2)$, respectively, on this scanning route. We also show the cross-sectional images of the isosurfaces at the $t_{\mathrm{F}} = 0$, $y = 0$, and $x = 0$ cuts in a similar manner to Figs. **S-1**(a)–(c). Unlike the rotational characteristic of E_{syn} shown in Figs. **S-1**(a)–(c), we observe that the electric field E_{syn} starts splitting into two temporal regions as the value of the CEP difference $|\Delta\varphi_{\mathrm{CE}}|$ increases to π. Figures **S-2**(d)–(f) show the intensity counterparts I_{syn} for Figs. **S-2**(a)–(c). The intensity envelope is split into two smaller OV pulses at $|\Delta\varphi_{\mathrm{CE}}|=\pi$, as shown in Fig. **S-2**(f). Interestingly, we can generate a double-pulsed sequence of the subcycle OV pulse by adjusting $(\varphi_{\mathrm{CE_S}}, \varphi_{\mathrm{CE_L}})$ to be $(-\pi/2, \pi/2)$, as shown in Figs. **S-2**(c) and (f), owing to the spatiotemporal coupling feature of the OV pulse. Here, we do not discuss the CEP difference among the three fields depicted in Figs. **S-2**(a)–(c) because it

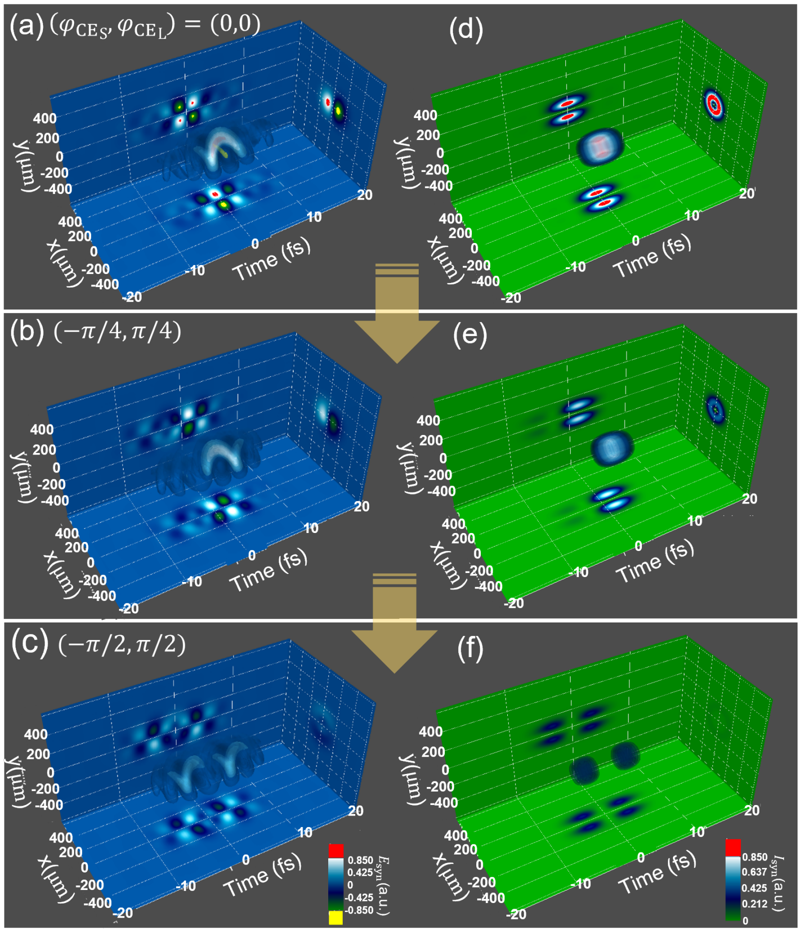

Fig. S-2. Isosurfaces of the electric field E_{syn} (a) with a CEP pair $(\varphi_{\mathrm{CE_S}}, \varphi_{\mathrm{CE_L}})$ of (0,0), (b) with a CEP pair of $(-\pi/4, \pi/4)$, and (c) with a CEP pair of $(-\pi/2, \pi/2)$. All the CEP pairs adopted in these calculations are on the scanning route satisfying $\varphi_{\mathrm{CE_S}} + \varphi_{\mathrm{CE_L}}$=0. (d)–(f) Isosurfaces of the spatiotemporal intensity profile I_{syn} of the synthesized field shown in Fig. **S-2**(a), shown in Fig. **S-2**(b), and shown in Fig. **S-2**(c), respectively.

is unclear how to determine the CEP origin of the pulse with a strongly modulated pulse envelope.

These results suggest that the temporal and spatial profiles of the synthesized waveform can be tailored simultaneously to form a complex spatiotemporal morphology if CEPs for each wavelength component of the OV pulses can be precisely controlled and carefully designed. In particular, the multiple OV pulses can be realized in a certain short period, as shown in Figs. **S-2**(c) and (f), which is advantageous for quantum information processing, for which a coherent pulse sequencer is highly desired [3]. We have reached one of the milestones by realizing such spatiotemporal control of the subcycle OV pulse in our experiment even though the absolute values of $\varphi_{\mathrm{CE_S}}$ and $\varphi_{\mathrm{CE_L}}$ are undetermined.

S-2. OUTPUT OF THE SAGNAC INTERFEROMETER: CHROMATIC ABERRATION

A. Formulation

The output field from an ideally configured Sagnac interferometer (SI), in which the path of the field propagating clockwise completely overlaps with that propagating counterclockwise, is written as [4]

$$\begin{aligned} E_{\text{out}}(x,y) =& (tt' + rr')E_{\text{in}}(x,y)\exp(ikL_{\text{R}}) \\ =& (tt - rr)E_{\text{in}}(x,y)\exp(ikL_{\text{R}}), \end{aligned} \tag{S-7}$$

where we denote t and r as the transmission amplitude and the reflection amplitude of a beam splitter (BS), respectively, when the light field $E_{\text{in}}(x,y)$ propagates from the air to the BS, and denote t' and r' as those when the light field propagates in the reverse direction. The optical path length of the ring is denoted as L_{R}. We assume the relations $t = t'$ and $r' = -r$ due to the π phase shift at the glass-to-air interface. The reflectance R and the transmittance T described in the main text are obtained as $R = rr$ and $T = tt$, respectively.

According to the configuration of the folding mirrors **M1**, **M2**, and **M3** in our SI depicted in Fig. 1(c) in the main text, which slightly deviates from the ideal configuration, the field after propagating clockwise in the SI experiences a vertical displacement of the field profile with the amount of d_y and a horizontal small angle deviation of θ_x ($\ll 1$) to the propagation direction of the ideal configuration. Because of the symmetry of the SI, the field after propagating counterclockwise should undergo the vertical displacement of $-d_y$ and the horizontal angle deviation of $-\theta_x$. As a result, the output field from our SI is expressed as

$$E_{\text{out}}(x,y) = T\,E_{\text{in}}(x, y-d_y)\exp(ik\theta_x x) - R\,E_{\text{in}}(x, y+d_y)\exp(-ik\theta_x x), \tag{S-8}$$

where the common phase factor $\exp[ik\cos(\theta_x)L_R]$ is omitted because it does not affect the interference between the first and the second terms on the right-hand side of Eq. (S-8), and we apply the approximation of $\sin\theta_x \simeq \theta_x$. In the experiment, the displacement d_y is controlled by vertically tilting **M1** and **M3** in opposite directions with an equal amount, while the angle deviation θ_x is adjusted by tilting **M2** in the horizontal direction. If the displacement d_y is much smaller than the waist size of an input Gaussian beam, the fields displaced with $\pm d_y$ can be expanded as a Taylor series, and then Eq. (S-8) can be rewritten as

$$\begin{aligned} &E_{\text{out}}(x,y) \\ &\approx \cos(k\theta_x x)\left[E_{\text{in}}(T-R) - d_y\frac{\partial E_{\text{in}}}{\partial y}(T+R) + \frac{d_y^2}{2!}\frac{\partial^2 E_{\text{in}}}{\partial y^2}(T-R) - \frac{d_y^3}{3!}\frac{\partial^3 E_{\text{in}}}{\partial y^3}(T+R) + \dots\right] \\ &\quad + i\sin(k\theta_x x)\left[E_{\text{in}}(T+R) - d_y\frac{\partial E_{\text{in}}}{\partial y}(T-R) + \frac{d_y^2}{2!}\frac{\partial^2 E_{\text{in}}}{\partial y^2}(T+R) - \frac{d_y^3}{3!}\frac{\partial^3 E_{\text{in}}}{\partial y^3}(T-R) + \dots\right]. \end{aligned} \tag{S-9}$$

We further impose the magnitude of θ_x to be so small that the condition $k\theta_x w_0 \ll 1$ is satisfied. Then, we approximate the sine and cosine functions of $k\theta_x x$ to the Taylor

expansion series up to second-order terms of $k\theta_x x$, i.e., $\cos(k\theta_x x) \approx 1 - (k\theta_x x)^2/2!$, and $\sin(k\theta_x x) \approx k\theta_x x$ because the maximum value of x is around w_0 at most. By substituting these Taylor series expansions into Eq. (S-9), we can express Eq. (S-9) as

$$E_{\text{out}}(x,y) \approx \left[1 - \frac{(k\theta_x x)^2}{2}\right]\left[E_{\text{in}}(T-R) - d_y \frac{\partial E_{\text{in}}}{\partial y}(T+R) + \frac{d_y^2}{2}\frac{\partial^2 E_{\text{in}}}{\partial y^2}(T-R)\right] + ik\theta_x x\left[E_{\text{in}}(T+R) - d_y \frac{\partial E_{\text{in}}}{\partial y}(T-R)\right], \tag{S-10}$$

where we omit the arguments in $E_{\text{in}}(x,y)$ to reduce the length of the formulae on the right-hand side of Eqs. (S-9) and (S-10). We finally take the terms up to the second order of small variables: $(d_y)^2$; $(k\theta_x x)^2$; $(d_y)(k\theta_x x)$ in Eq. (S-10).

When the transmittance and reflectance of the BS are the same, we obtain

$$E_{\text{out}}(x,y) = -\, d_y\left(\frac{\partial E_{\text{in}}(x,y)}{\partial y}\right) + i(k\theta_x x)E_{\text{in}}(x,y), \tag{S-11}$$

by substituting $T = R$ in Eq. (S-10), where we assume that the BS is lossless; and thus, $T + R = 1$. Eq. (S-11) was derived by Damzen et al. [4].

Considering that the input field is a Gaussian beam in a far field, and thus assuming that the wavefront curvature is so large that the phase front of the input beam may be regarded as a plane perpendicular to the propagation axis z, $E_{\text{in}}(x,y)$ may be written as $E_{\text{in}}(x,y) = \sqrt{2/[\pi w^2(\lambda)]}\mathrm{e}^{-\frac{x^2+y^2}{w^2(\lambda)}}$, where we assume that the beam waist $w(\lambda)$ depends on the wavelength λ to describe the chromatic aberration. The electric field $E_{\text{in}}(x,y)$ is normalized so as to satisfy $\int dxdy|E_{\text{in}}(x,y)|^2 = 1$. By substituting this Gaussian field into Eq. (S-11), we can rewrite the output field as

$$E_{\text{out}}(x,y) = 2i\sqrt{\frac{2}{\pi w^2(\lambda)}}\frac{1}{w(\lambda)}\left[\alpha_x(\lambda)x - i\beta_y(\lambda)y\right]\mathrm{e}^{-\frac{x^2+y^2}{w^2(\lambda)}}, \tag{S-12}$$

where we define $\alpha_x(\lambda) \equiv kw(\lambda)\theta_x/2 = \pi w(\lambda)\theta_x/\lambda$ and $\beta_y(\lambda) \equiv d_y/w(\lambda)$, as described in **Results** in the main text. It is natural to impose the beam waist to be proportional to the wavelength; thus, we assume the relation $w(\lambda) = (\lambda/\lambda_{\mathrm{r}})\, w_{\mathrm{r}}$, where w_{r} is the beam waist for the reference wavelength λ_{r}. Under this constraint of the wavelength scaling for the beam waist, we find the relation $\alpha_x(\lambda) = \alpha_x(\lambda_{\mathrm{r}})$, namely, the parameter α_x is independent of λ. Therefore, we omit the argument of α_x in the following. We also add the subscript $_{\mathrm{r}}$ to notations to describe the quantities at the reference wavelength.

When the condition $\alpha_x{=}\beta_{y_{\mathrm{r}}}$ is satisfied at the reference wavelength, the output field described in Eq. (S-12) is transformed to

$$\begin{aligned} E_{\text{out}_{\mathrm{r}}}(x,y) &= E_{\text{out}_{\mathrm{r}}}(r\cos\phi, r\sin\phi) \\ &= i\sqrt{2}\left(\frac{d_y}{w_{\mathrm{r}}}\right)\mathrm{LG}_{\mathrm{r},p=0}^{\ell=+1}(r,\phi,z=0), \end{aligned} \tag{S-13}$$

where $\mathrm{LG}_{\mathrm{r},p=0}^{\ell=+1}(r,\phi,z=0)$ is obtained by substituting $p = 0$, $\ell = +1$, $z = 0$, and replacing w_0 with w_{r} in Eq. (S-1). Therefore, the output field at the reference wavelength becomes

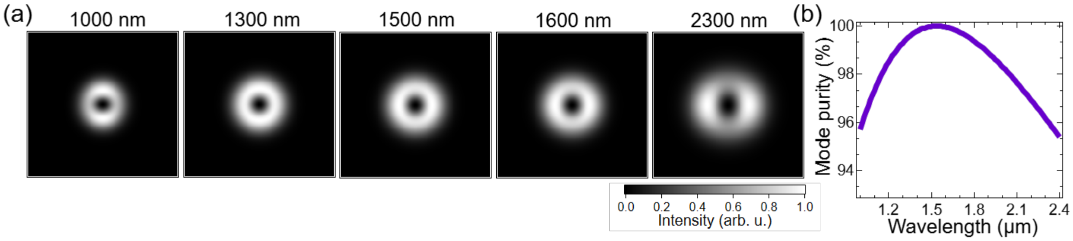

Fig. S-3. (a) Intensity profiles of the output field $E_{\text{out}}(x, y)$ from SI, which are calculated using Eq. (S-12), at wavelengths of 1000 nm (leftmost panel), 1300 nm (second left panel), 1500 nm (center panel), 1600 nm (second right panel), and 2300 nm (rightmost panel). The displacement d_y and the angle deviation θ_x are fixed to generate an ideal OV beam at the reference wavelength of 1500 nm. (b) Mode purity $\mathsf{p_u}$ calculated using Eq. (S-16).

an ideal OV field with $\ell = +1$. Because we utilize the relationship between the Cartesian coordinates and the cylindrical coordinates expressed as $x - iy = r\text{e}^{-i\phi}$ to derive Eq. (S-13), we can change the sign of the topological charge ℓ by changing the sign of one of the parameters α_x or β_{y_r}. We adjust the vertical tilts of mirror **M1** and mirror **M3** to switch the vertical displacement to the opposite direction, for example, the sign of β_{y_r} changes to negative. As a result, we obtain the OV field with $\ell = -1$ expressed as

$$E_{\text{out}_\text{r}}(r\cos\phi, r\sin\phi) = i\sqrt{2}\left(\frac{d_y}{w_\text{r}}\right)\text{LG}^{\ell=-1}_{\text{r},p=0}(r, \phi, z = 0), \tag{S-14}$$

owing to the relation $x + iy = r\text{e}^{i\phi}$. Therefore, we can generate the OV field with $\ell = +1$ or $\ell = -1$ at a reference wavelength from the SI under the appropriate condition of the transmittance/reflectance of the BS and the alignment of folding mirrors **M1**, **M2**, and **M3**.

B. Chromatic aberration

When the wavelength is deviated from λ_r, the output field from the SI does not coincide with the formula on the right-hand side of Eq. (S-13). Thus, we adopt Eq. (S-12) to calculate the output field and examine how the wavelength deviation distorts the output field from the ideal OV field. Note that we still assume $T = R$ of the BS in this calculation.

For the readers' convenience, we show again the calculation results of $|E_{\text{out}}(x, y)|^2$ as beam intensity profiles of the output fields at wavelengths of 1000 nm, 1300 nm, 1500 nm, 1600 nm, and 2300 nm in the five panels of Fig. **S-3**(a), which are the same as the panels in Fig. 2(c) in the main text. In the calculations, we fix λ_r =1500 nm, α_x=β_{y_r} =0.4, and θ_x =0.2 mrad, so that we have to impose the beam waist at λ_r, w_r ≒0.955 mm and the vertical displacement d_y ≒0.382 mm.

In Fig. **S-3**(a), the doughnut-shaped structure with a homogeneous intensity distribution along the azimuth angle is observed in the beam profile at 1500 nm, as we expected. As the wavelength becomes longer from 1500 nm to 2300 nm, the beam profile is continuously deformed into an ellipsoidal shape with the horizontal main axis, and the intensity on the horizontal sides gradually increases. On the other hand, as the wavelength becomes shorter from 1500 nm to 1000 nm, the beam profile is continuously deformed into an ellipsoidal

shape with the vertical main axis, and the intensity on the vertical sides gradually increases. Note that the radius of the main axis of the beam profile itself increases when the wavelength increases. This is due to the assumption that $w(\lambda) = (\lambda/\lambda_{\rm r})\, w_{\rm r}$, which we impose on the beam waist. These characteristics of the beam profile deformation are very similar to those observed in the experimentally recorded beam profile images shown in Fig. 2(a) in the main text. Therefore, we conclude that the beam profile deformation seen in Fig. 2(a) in the main text mainly originates from the chromatic aberration intrinsic in the mode conversion process using the SI.

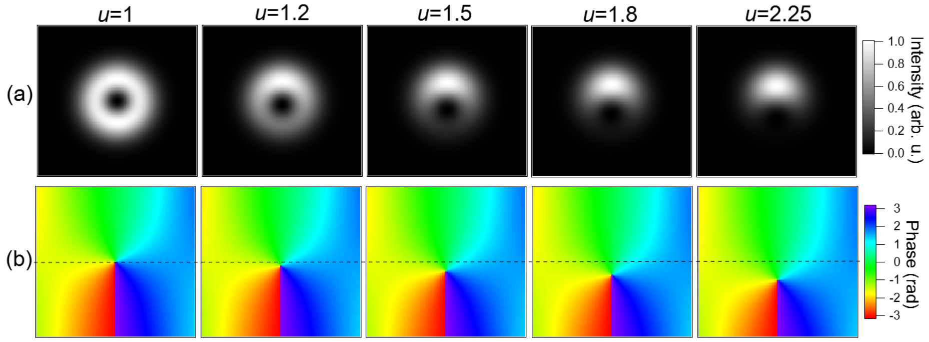

Fig. S-4. (a) Intensity profiles of the output field $|E_{\rm out}(u; x, y)|^2$ calculated using Eq. (S-17) with $u = \frac{T}{R}$ =1 (leftmost panel), 1.2 (second left panel), 1.5 (center panel), 1.8 (second right panel), and 2.25 (rightmost panel). (b) Argument of the output field $\arg\{E_{\rm out}(u; x, y)\}$ with u =1 (leftmost panel), 1.2 (second left panel), 1.5 (center panel), 1.8 (second right panel), and 2.25 (rightmost panel).

Nevertheless, we show in the following that the SI output field is still suitable for approximating the OV field even when the wavelength deviates to the shortest or longest side in the spectral range of the subcycle pulse. To this end, we rewrite Eq. (S-12) as

$$\begin{aligned} &E_{\rm out}(r\cos\phi, r\sin\phi) \\ &\quad = i\sqrt{2}\left[\frac{\alpha_x+\beta_y(\lambda)}{2}\mathrm{LG}_{p=0}^{\ell=1}(r,\phi,z=0) + \frac{\alpha_x-\beta_y(\lambda)}{2}\mathrm{LG}_{p=0}^{\ell=-1}(r,\phi,z=0)\right], \end{aligned} \tag{S-15}$$

where $\mathrm{LG}_p^\ell(r,\phi,z=0)$ is obtained by substituting $z = 0$ and replacing w_0 with $w(\lambda)$ in $\mathrm{LG}_p^\ell(r,\phi,z)$ shown in Eq. (S-1). Therefore, the output field from the SI includes the LG mode function with $\ell = -1$, and it is reasonable to define the purity $\mathsf{p_u}$ of the output field as the intensity ratio of the LG mode function with $\ell = 1$ to the total fields, namely,

$$\mathsf{p_u} \equiv \frac{\{\alpha_x+\beta_y(\lambda)\}^2}{\{\alpha_x+\beta_y(\lambda)\}^2+\{\alpha_x-\beta_y(\lambda)\}^2} = \frac{(\lambda+\lambda_{\rm r})^2}{2(\lambda^2+\lambda_{\rm r}^2)}, \tag{S-16}$$

where we use the relations $\alpha_x = \beta_{y_{\rm r}}$ and $w(\lambda) = (\lambda/\lambda_{\rm r})\, w_{\rm r}$ to obtain the rightmost formula in Eq. (S-16).

The wavelength dependence of the mode purity $\mathsf{p_u}$ calculated using Eq. (S-16) is depicted as a solid curve in Fig. **S-3**(b). In this calculation, we use the same parameters adopted for the calculation of the beam profiles shown in Fig. **S-3**(a). We identify from this figure that the mode purity is approximately more than 95% in the entire range of the wavelength of the subcycle pulse; and therefore, we conclude that the characteristic of the OV field is sufficiently maintained in this wavelength range.

C. Effect of T/R ratio

In the previous sections, we assumed that the transmittance T and the reflectance R of the BS are completely the same. In this section, we examine the effect of the ratio $u = T/R$ on the beam profile at the reference wavelength λ_r. To evaluate how the output field from the SI is deformed with the alteration of u, we transform Eq. (S-10) into the following form by using $E_{\mathrm{out_r}}(x, y)$ defined in Eq. (S-13).

$$E_\mathrm{out}(u; x, y) = E_{\mathrm{out_r}}(x, y) + \frac{u-1}{u+1}\left[E_\mathrm{in}(x, y) - \frac{(k\theta_x x)^2}{2}E_\mathrm{in}(x, y) - ik\theta_x x d_y \frac{\partial E_\mathrm{in}(x, y)}{\partial y} + \frac{d_y^2}{2}\frac{\partial^2 E_\mathrm{in}(x, y)}{\partial y^2}\right]. \tag{S-17}$$

We assume again the lossless BS satisfying $T + R = 1$ to derive Eq. (S-17). Here, we also take the terms up to the second order of small variables: $(d_y)^2$, $(k\theta_x x)^2$, and $(d_y)(k\theta_x x)$ in Eq. (S-17).

We can clearly see from Eq. (S-17) that $E_\mathrm{out}(u; x, y)$ deviates from the exact LG mode $E_{\mathrm{out_r}}(x, y)$ with an additional term proportional to $(u - 1)/(u + 1)$ except for $u = T/R = 1$. The image of the magnitude square of $E_\mathrm{out}(u; x, y)$ was deformed from the exact LG mode to a different mode by increasing u from 1, as shown in Fig. **S-4**(a). Upon the increase in $u = T/R$, the magnitude peak gradually shifts upward ($+y$-direction) in this figure, whereas the phase singular point moves downward ($-y$-direction), as shown in Fig. **S-4**(b). Note that we specify the y-center point of the phase diagrams as a dashed line in each panel of Fig. **S-4**(b). The dielectric coats on both sides of the BS we used in the experiment are designed to satisfy the condition $0.95 \leq T/R \leq 1.1$ in the entire wavelength range of the subcycle pulse. Therefore, we conclude that the mode deviation originating from the deviation of the T/R value from 1 is reasonably small.

S-3. PHASE CHARACTERIZATION WITH FERMAT'S SPIRAL

To characterize the phase fidelity of the experimental results, we have extracted the phase distribution contour from the experimental spiral fringes [5] and compared them with that of the simulated results in the main text. This method is more convenient for the evaluation of the phase front of the OV pulse than the conventional phase retrieval method by utilizing the Fourier transform of the spatial interference fringes [6] because special care about the helical structure of the wavefront and the phase singularity of the OV pulse must be taken into account in the conventional method. In this section, we explain how to obtain the model formulae used in this calculation.

First, we have to refer to Eq. (S-3) with the phase term of the wavefront, because the spiral fringes observed in Figs. 2(d) and 5(e) in the main text originate from the spatial interference between the OV pulse and the reference Gaussian beam with different curvatures of the wavefronts. We define the following electric field of the OV pulse as $E^\mathrm{v}(r, \phi, z, t)$ and that of the Gaussian beam as $E^\mathrm{g}(r, \phi, z, t)$,

$$E^\mathrm{v}(r, \phi, z, t) \equiv \widetilde{u}^{\,\ell}_{\mathrm{v}\,p=0}(r, \phi, z)\mathrm{e}^{-ik_\mathrm{v}\frac{r^2}{2R_\mathrm{v}(z)} - i\Theta^{\ell}_{p=0,\mathrm{v}}(z) - i\ell\phi}\varepsilon_\mathrm{v}(t - \frac{z}{c})\mathrm{e}^{i\omega_\mathrm{v}t - ik_\mathrm{v}z}\mathrm{e}^{i\varphi_{\mathrm{CE_v}}}, \tag{S-18}$$

$$E^\mathrm{g}(r, \phi, z, t) \equiv \widetilde{u}^{\,\ell=0}_{\mathrm{g}\,p=0}(r, \phi, z)\mathrm{e}^{-ik_\mathrm{g}\frac{r^2}{2R_\mathrm{g}(z)} - i\Theta^{\ell=0}_{p=0,\mathrm{g}}(z)}\varepsilon_\mathrm{g}(t - \frac{z}{c})\mathrm{e}^{i\omega_\mathrm{g}t - ik_\mathrm{g}z}\mathrm{e}^{i\varphi_{\mathrm{CE_g}}}, \tag{S-19}$$

on the detector plane $(r\cos\phi, r\sin\phi)$ situated at the propagation distance z, in accordance with Eq. (S-3). We assume that the two fields propagate with the same carrier angular frequency of $\omega_{\mathrm{v}} = \omega_{\mathrm{g}} = \omega_c$ ($k_{\mathrm{v}} = k_{\mathrm{g}} = k_c$) to simulate the f-f interference experiments demonstrated in the third section of **Results** in the main text. The beam radius $w_{\mathrm{v}}(z)$, the wavefront curvature $R_{\mathrm{v}}(z)$, the Gouy phase $\Theta^{\ell}_{p=0,\mathrm{v}}(z)$, and the CEP $\varphi_{\mathrm{CE_v}}$ of the OV pulse may differ from the Gaussian beam ($w_{\mathrm{g}}(z)$, $R_{\mathrm{g}}(z)$, $\Theta^{\ell=0}_{p=0,\mathrm{g}}(z)$, and $\varphi_{\mathrm{CE_g}}$), respectively. We omit the argument z of these quantities in the following because the detector plane is fixed. We also discard the subscript $p = 0$.

The image on the detector plane $I(r,\phi)$ is given by $I(r,\phi) = \int dt |E^{\mathrm{v}}(r,\phi,z,t) + E^{\mathrm{g}}(r,\phi,z,t)|^2$. As a result, we obtain the following formula:

$$I(r,\phi) = I_{\mathrm{v}} |\widetilde{u_{\mathrm{v}}}^{\ell}(r,\phi)|^2 + I_{\mathrm{g}} |\widetilde{u_{\mathrm{g}}}^{0}(r,\phi)|^2 + 2I_{\mathrm{vg}} \widetilde{u_{\mathrm{v}}}^{\ell}(r,\phi) \widetilde{u_{\mathrm{g}}}^{0}(r,\phi) \cos\left[\frac{k_c}{2\Delta R_{\mathrm{vg}}} r^2 + \ell\phi + \Delta\Theta^{\ell}_{\mathrm{vg}} - \Delta\varphi_{\mathrm{CE_{vg}}}\right], \quad \text{(S-20)}$$

where we define $I_{\mathrm{v}} \equiv \int dt \varepsilon^2_{\mathrm{v}}(t)$, $I_{\mathrm{g}} \equiv \int dt \varepsilon^2_{\mathrm{g}}(t)$, and $I_{\mathrm{vg}} \equiv \int dt \varepsilon_{\mathrm{v}}(t) \varepsilon_{\mathrm{g}}(t)$. By assuming that the temporal envelope of the Gaussian beam is proportional to that of the OV pulse, we apply the relations $I_{\mathrm{v}} = I_0(1-\xi)$, $I_{\mathrm{g}} = I_0\xi$, and $I_{\mathrm{vg}} = I_0\sqrt{(1-\xi)\xi}$ $(0 \le \xi \le 1)$ to Eq. (S-20). The parameter ξ specifies the weighting of the Gaussian beam. In addition, we introduce the following notations: $(\Delta R_{\mathrm{vg}})^{-1} \equiv R^{-1}_{\mathrm{v}} - R^{-1}_{\mathrm{g}}$, $\Delta\Theta^{\ell}_{\mathrm{vg}} \equiv \Theta^{\ell}_{\mathrm{v}} - \Theta^{0}_{\mathrm{g}}$, and $\Delta\varphi_{\mathrm{CE_{vg}}} \equiv \varphi_{\mathrm{CE_v}} - \varphi_{\mathrm{CE_g}}$. The real amplitude $\widetilde{u_{\mathrm{v}}}^{\ell}(r,\phi)$ is obtained by substituting $p = 0$ and replacing $w(z)$ with w_{v} in Eq. (S-2), and the real amplitude $\widetilde{u_{\mathrm{g}}}^{0}(r,\phi)$ is obtained similarly.

We intend to extract the edge of the spiral fringe as a contour plot, which is expressed as variables (r,ϕ) satisfying

$$\cos\left[\frac{k_c}{2\Delta R_{\mathrm{vg}}} r^2 + \ell\phi + \Delta\Theta^{\ell}_{\mathrm{vg}} - \Delta\varphi_{\mathrm{CE_{vg}}}\right] = 0. \quad \text{(S-21)}$$

This equation is equivalent to

$$\frac{k_c}{2\Delta R_{\mathrm{vg}}} r^2 + \ell\phi + \Delta\Theta^{\ell}_{\mathrm{vg}} - \Delta\varphi_{\mathrm{CE_{vg}}} = \left(n + \frac{1}{2}\right)\pi \quad (n = 0, \pm1, \pm2, ...), \quad \text{(S-22)}$$

and has the same form as a Fermat's spiral [5],

$$\phi = Ar^2 + B, \quad \text{(S-23)}$$

where the coefficients A and B are given by

$$A = -\frac{\ell^{-1} k_c}{2\Delta R_{\mathrm{vg}}}, \quad \text{(S-24)}$$

$$B = \ell^{-1}\left[\left(n + \frac{1}{2}\right)\pi - \Delta\Theta^{\ell}_{\mathrm{vg}} + \Delta\varphi_{\mathrm{CE_{vg}}}\right], \quad \text{(S-25)}$$

and the coefficient B expresses the offset azimuth angle. We adopt Eq. (S-20) to simulate the spiral image of the fringe and apply Eq. (S-23) with Eqs. (S-24) and (S-25) to fit the contours extracted from the spiral fringes in the experiment. Note that $\Delta\varphi_{\mathrm{CE_{vg}}}$ does not change when scanning the CEP because $\varphi_{\mathrm{CE_v}}$ and $\varphi_{\mathrm{CE_g}}$ both emerge from the same wavelength component of the fundamental (f) field.

To characterize the phase of experimental results, we first extract the spiral contour from the measurements, as indicated by dots in Fig. 2(d) in the main text, by the Canny edge method [7]. Then, we change the contour coordinate from the Cartesian system to the cylindrical system with the appropriate unwrap of the azimuth angle ϕ, shown as dots in Fig. 2(f) in the main text. This contour on the r–ϕ plane is fitted by Eq. (S-23). The resultant fitting parameters A and B used for drawing the fitting curves in the first, second, third, fourth, and fifth panels in Fig. 2(f) in the main text are listed in **Table S-1**. The images in the five panels of Fig. 2(e) are calculated by substituting the resultant fitting parameters A and B into Eq. (S-20) via Eqs. (S-24) and (S-25), namely, $\frac{k_c}{2\Delta R_{\mathrm{vg}}} = -A$ and $\Delta\Theta^{\ell}_{\mathrm{vg}} - \Delta\varphi_{\mathrm{CE_{vg}}} = -B + \frac{\pi}{2}$ because $\ell = 1$ and $n = 0$. According to Eq. (S-23), the azimuth angle increases upon the increase in the radius when the coefficient A is positive. Therefore, the spiral winds around the center counterclockwise in this case. Here, the coefficient A is negative, so the spiral in each panel of Fig. 2(c) winds clockwise.

Table S-1. Fitting results for A and B

Wavelength (nm)	A (mm^{-2})	B (rad)
1000	−21.33	1.03
1300	−16.78	0.88
1500	−14.37	2.48
1600	−13.46	3.20
2300	−9.20	2.52

The RMS error (ϕ_{rms}) and R-squared value (ϕ_{R^2}) of the ϕ-fit are defined as $\phi_{\mathrm{rms}} \equiv \sqrt{S_{\mathrm{res}}/N}$ and $\phi_{\mathrm{R}^2} \equiv 1 - S_{\mathrm{res}}/S_{\mathrm{tot}}$, respectively, where $S_{\mathrm{res}} \equiv \sum_{i=1}^{N} (\phi_{\mathrm{exp},i} - \phi_{\mathrm{fit},i})^2$ is the residual sum of squares and $S_{\mathrm{tot}} \equiv \sum_{i=1}^{N} (\phi_{\mathrm{exp},i} - \overline{\phi}_{\mathrm{exp}})^2$ is the total sum of squares. We denote the ith experimental data point as $(r_{\mathrm{exp},i}, \phi_{\mathrm{exp},i})$ and the azimuth angle resulting from the fit as $\phi_{\mathrm{fit},i} \equiv A r^2_{\mathrm{exp},i} + B$ by using the fitting results for A and B listed in **Table S-1**. We also define the average of the N experimental data points as $\overline{\phi}_{\mathrm{exp}}$. Generally, the RMS error helps us quantify the residues and the R-squared value enables us to measure the strength of the relationship between the observed values in the experiment and the modeled values in the fit. Thus, we have evaluated the accuracy of the azimuth angle ϕ with these quantities obtained from the spiral interference fringes to show the fidelity of the OV pulse in the experiment, even though the spiral interference fringes are originally utilized to show the nature of the helical wavefront.

S-4. CHARACTERISTIC OF THE SPIRAL INTERFERENCE FRINGE IN 2D *f*-2*f* INTERFEROMETRY

In this section, we explain the reason why the spiral interference fringe obtained by 2D f-$2f$ interferometry rotates upon changing CEP. In the setup of the 2D f-$2f$ interferometry, the Gaussian beam is frequency-doubled in a nonlinear crystal, and we can assume that the amplitude of the generated second-harmonic (SH) beam is proportional to the square of the input fundamental Gaussian beam. By considering that the square of the real Gaussian amplitude $\{\widetilde{u}_{\mathrm{g}p=0}^{\ell=0}(r, \phi, z)\}^2$ [8] in Eq. (S-19) can also be expressed as another real Gaussian amplitude, which we define as $\widetilde{u_{\mathrm{SH}}}_{p=0}^{\ell=0}(r, \phi, z)$, we can obtain the amplitude of the SH beam

$E^{\mathrm{SH}}(r,\phi,z,t)$ by solving the following formula:

$$E^{\mathrm{SH}}(r,\phi,z,t) \propto \widetilde{u_{\mathrm{SH}}}_{p=0}^{\ell=0}(r,\phi,z)\mathrm{e}^{-i2k_{\mathrm{g}}\frac{r^2}{2R_{\mathrm{g}}(z)}-2i\Theta_{p=0,\mathrm{g}}^{\ell=0}(z)}\varepsilon_{\mathrm{g}}^{2}(t-\frac{z}{c})\mathrm{e}^{2i\omega_{\mathrm{g}}t-2ik_{\mathrm{g}}z}\mathrm{e}^{2i\varphi_{\mathrm{CE_g}}}, \quad \text{(S-26)}$$

where the real amplitude $\widetilde{u_{\mathrm{SH}}}_{p=0}^{\ell=0}(r,\phi,z)$ is obtained by substituting $p=0$, $\ell=0$, and replacing $w(z)$ with w_{SH} in Eq. (S-2). We define the beam radius of the SH Gaussian beam to be w_{SH} at the detector plane, as was assumed in **Sec. S-3**. In the experiment, we insert a bandpass filter in front of the IR CCD camera to only detect the spectral region where the wavelength component at the shortest edge of the fundamental (f) OV pulse overlaps the wavelength component at the longest edge of the SH ($2f$) Gaussian beam. Under this experimental condition, we can impose $k_{\mathrm{v}}=2k_{\mathrm{g}}\equiv k_{\mathrm{o}}$ and $\omega_{\mathrm{v}}=2\omega_{\mathrm{g}}\equiv\omega_{\mathrm{o}}$ in Eqs. (S-18) and (S-26). Therefore, the image on the detector plane $I_{f2f}(r,\phi)=\int dt|E^{\mathrm{v}}(r,\phi,z,t)+E^{\mathrm{SH}}(r,\phi,z,t)|^2$ is given by

$$\begin{aligned} I_{f2f}(r,\phi) &= I_{\mathrm{v}}|\widetilde{u_{\mathrm{v}}}^{\ell}(r,\phi)|^2 + I_{\mathrm{SH}}|\widetilde{u_{\mathrm{SH}}}^{0}(r,\phi)|^2 \\ &\quad + 2I_{\mathrm{vSH}}\widetilde{u_{\mathrm{v}}}^{\ell}(r,\phi)\widetilde{u_{\mathrm{SH}}}^{0}(r,\phi)\cos\left[\frac{k_{\mathrm{o}}}{2\Delta R_{\mathrm{vg}}}r^2+\ell\phi+\Delta\Theta_{\mathrm{vSH}}^{\ell}-\left\{\varphi_{\mathrm{CE_v}}-2\varphi_{\mathrm{CE_g}}\right\}\right], \end{aligned} \quad \text{(S-27)}$$

where we define $I_{\mathrm{SH}}\equiv\int dt\varepsilon_{\mathrm{g}}^{4}(t)$ and $I_{\mathrm{vSH}}\equiv\int dt\varepsilon_{\mathrm{v}}(t)\varepsilon_{\mathrm{g}}^{2}(t)$. We assume that the temporal envelope of the SH Gaussian beam, which is equivalent to the square of the temporal envelope of the fundamental Gaussian beam, is proportional to that of the OV pulse because they pass through the common bandpass filter, resulting in the same small bandwidth. With this assumption, we apply the relations $I_{\mathrm{v}}=I_0(1-\zeta)$, $I_{\mathrm{SH}}=I_0\,\zeta$, and $I_{\mathrm{vSH}}=I_0\sqrt{(1-\zeta)\zeta}$ $(0\le\zeta\le1)$ to Eq. (S-27), where the coefficient ζ specifies the weighting of the SH Gaussian beam.

Because the CEP of the OV pulse $\varphi_{\mathrm{CE_v}}$ is involved in the shorter side of the spectral region, we can substitute $\varphi_{\mathrm{CE_v}}=\varphi_{\mathrm{CE_S}}=\delta\varphi_{\mathrm{CE_S}}+\varphi_{\mathrm{CE_{S_0}}}$, as mentioned in the last section of **Results** in the main text. We can also justify the relation $\varphi_{\mathrm{CE_g}}=\varphi_{\mathrm{CE_L}}=\delta\varphi_{\mathrm{CE_L}}+\varphi_{\mathrm{CE_{L_0}}}$ for a similar reason. To find the evolution of the spiral fringe, we focus on the contour as a sequence of local maxima of the fringe derived by requiring that the argument of the cosine function in Eq. (S-27) is 0. The resulting equation of the contour exhibits the form of a Fermat's spiral shown in Eq. (S-23), but the coefficients A and B are somewhat different from those described in Eqs. (S-24) and (S-25), and they are given as

$$A=-\frac{\ell^{-1}k_{\mathrm{o}}}{2\Delta R_{\mathrm{vg}}}, \quad \text{(S-28)}$$

$$\begin{aligned} B &= \ell^{-1}\left(\varphi_{\mathrm{CE_v}}-2\varphi_{\mathrm{CE_g}}-\Delta\Theta_{\mathrm{vSH}}^{\ell}\right) \\ &= \ell^{-1}\left(\delta\varphi_{\mathrm{CE_S}}-2\delta\varphi_{\mathrm{CE_L}}+\varphi_{\mathrm{o}}\right), \end{aligned} \quad \text{(S-29)}$$

where we define $\varphi_{\mathrm{o}}\equiv-\Delta\Theta_{\mathrm{vSH}}^{\ell}+\varphi_{\mathrm{CE_{S_0}}}-2\varphi_{\mathrm{CE_{L_0}}}$. We can recognize from Eq. (S-29) that the offset azimuth angle B is varied by the CEP shift of $\delta\varphi_{\mathrm{CE_S}}$ applied from the AOPDF controlling the short-wavelength component and that of $\delta\varphi_{\mathrm{CE_L}}$ applied from the AOPDF controlling the long-wavelength component, whereas the coefficient A remains unchanged. This is the reason why the spiral interference fringe rotates without deforming its structure under the modulations of $\delta\varphi_{\mathrm{CE_S}}$ and $\delta\varphi_{\mathrm{CE_L}}$. The direction of the rotation is reversed by switching the sign of ℓ.

To calculate the 2D f-$2f$ spiral interference fringes shown in the first and third rows in the panels of Fig. 5(e) in the main text, we use Eq. (S-27) with the parameters $\ell = 1$, $\zeta \fallingdotseq 0.5$, $\frac{k_o}{2\Delta R_{vg}} \fallingdotseq 13.5\ \text{mm}^{-2}$, $w_v \fallingdotseq 1$ mm, and $w_{SH} \fallingdotseq 3$ mm.

We summarize the modulation functions for $\delta\varphi_{CE_S}$ and $\delta\varphi_{CE_L}$ used for the CEP scan in Fig. 5(b) in the main text and three videos in **Table S-2**, where time is denoted as t' and period as $T(\sim 50$ s).

Table S-2. Modulation functions for $\delta\varphi_{CE_S}$ and $\delta\varphi_{CE_L}$.

Videos	$\delta\varphi_{CE_S}$	$\delta\varphi_{CE_L}$
***Video 1**	$12\sin^{-1}[\sin(2\pi t'/T)]$	$12\sin^{-1}[\sin(2\pi t'/T)]$
**Video 2**	$-6\pi\sin(2\pi t'/T)$	$6\pi\sin(2\pi t'/T)$
**Vidoe 3**	$12\sin^{-1}[\sin(4\pi t'/T)]$	$12\sin^{-1}[\sin(2\pi t'/T)]$

*The functions in this row are also applied to obtain Fig. 5(b) in the main text.

---

[1] L. Allen, M. W. Beijersbergen, R. Spreeuw, and J. Woerdman, Orbital angular momentum of light and the transformation of Laguerre–Gaussian laser modes, Physical Review A **45**, 8185 (1992).

[2] Y.-C. Lin, K. Midorikawa, and Y. Nabekawa, Carrier-envelope phase control of synthesized waveforms with two acousto-optic programmable dispersive filters, Optics Express **30**, 10818 (2022).

[3] M. Hosseini, B. M. Sparkes, G. Hétet, J. J. Longdell, P. K. Lam, and B. C. Buchler, Coherent optical pulse sequencer for quantum applications, Nature **461**, 241 (2009).

[4] M. J. Damzen, W. R. Kerridge-Johns, and J. Geberbauer, Vortex mode transformation interferometry, Journal of Optics **22**, 015604 (2019).

[5] E. Frączek, A. Popiołek-Masajada, and S. Szczepaniak, Characterization of the vortex beam by Fermat's spiral, in *Photonics*, Vol. 7 (MDPI, 2020) p. 102.

[6] M. Takeda, H. Ina, and S. Kobayashi, Fourier-transform method of fringe-pattern analysis for computer-based topography and interferometry, J. of Opt. Soc. Am. **72**, 156 (1982).

[7] J. Canny, A computational approach to edge detection, IEEE Transactions on pattern analysis and machine intelligence **PAMI-8**, 679 (1986).

[8] Y.-C. Lin, K.-F. Huang, and Y.-F. Chen, The formation of quasi-nondiffracting focused beams with second-harmonic generation of flower Laguerre–Gaussian modes, Laser Physics **23**, 115405 (2013).
